# MGH: a genome hub for the medicinal plant maca (*Lepidium meyenii*)

**DOI:** 10.1093/database/bay113

**Published:** 2018-10-19

**Authors:** Junhao Chen, Jiawei Zhang, Meigui Lin, Wei Dong, Xinyue Guo, Yang Dong, Zhengjia Wang, Liangsheng Zhang, Fei Chen

**Affiliations:** 1 State Key Laboratory of Subtropical Silviculture, School of Forestry and Biotechnology, Zhejiang A&F University, Hangzhou, China; 2 State Key Laboratory of Ecological Pest Control for Fujian and Taiwan Crops, Key Laboratory of Genetics, Breeding and Multiple Utilization of Corps, Ministry of Education, Fujian Provincial Key Laboratory of Haixia Applied Plant Systems Biology, Fujian Agriculture and Forestry University, Fuzhou, China; 3 Biological Big Data College, Yunnan Agricultural University, Kunming, China; 4 BGI-Shenzhen, Shenzhen, Guangdong, China; 5 National & Local Joint Engineering Research Center on Germplasm Utilization & Innovation of Chinese Medicinal Materials in Southwestern China, Kunming, China

## Abstract

Maca (*Lepidium meyenii*), a Brassicaceae herb plant originated from Andean mountains, has attracted wide interests due to its unique health benefits in reproduction and fertility. Because of its adaptation to the 4000 m high-altitude harsh environment, maca is attracting more and more attention from both crop breeders and basic biologists. After our previous release of the maca genome sequence, there’s a growing need to store, query, analyze and integrate various maca resources efficiently. Here, we created Maca Genome Hub (MGH), a genomics and genetics database of maca. Currently, the MGH V1.0 harbors the genome sequence, predicted coding sequences and protein sequences, various annotations, markers and expression data. For the maca research community, we also provided the publications, researchers and related news. MGH is designed to enable users’ easy access to analyze, retrieve and visualize the genomic or genetic information through a series of online tools, including the Basic Local Alignment Search Tool, the JBrowse, the query system, the synteny tool and the data downloads. These integrated heterogeneous data, tools and interfaces in MGH allow efficient mining of the latest genomics and genetics data. We hope that MGH will accelerate the research and development in maca.

## Introduction

Maca (*Lepidium meyenii,* Brassicaceae) is native to the Central Andes mountains. Since the domestication of maca started ∼2000 years ago in central Peru as food and medicine (1), nowadays maca have been introduced to many places outside Latin America and have been widely cultivated in China as a `Peruvian Viagra’ or `herbal Viagra’, referring to its functions in treating the sexual dysfunctions (2, 3). Maca is cultivated for its large and fleshy hypocotyl, which grows into a pear shape with diverse colors such as gold, red, purple, green and black (4).

Maca can adapt to very harsh regions and to extremely high altitudes up to 4500 m. The *Lepidium* genus (Figure S1), belonging to Brassicaceae lineage I (Figure S2), covers 230 species distributed widely across temperate, subtropical and cold regions. This genus arose as diploid species, with *n* = 8 chromosomes (source: http://ccdb.tau.ac.il/search/ (5); Figure S3). Maca contains an octoploid chromosome organization 2*n* = 8*x* = 64. It was proposed that polyploidization, particularly the allopolyploidization, facilitated its colonization of new environments (6). The hypocotyl of maca has been demonstrated to have an effect on sexual function, spermatogenesis, female reproductive function, memory, depression and anxiety and energy as well as effects on benign prostatic hyperplasia, osteoporosis and metabolic syndrome (7, 8). Its antioxidant and anti-aging effects were also well documented (1).

The genome of a domesticated maca cultivated at 4200 meter highlands (Figure S4) was sequenced using Illumina HiSeq 2500 platform. The 361 Gb raw reads covered 482-fold of the 751 Mb genome. The assembled genome scaffolds had a total length of 743.2 Mb (Figure S5). Core eukaryotic genes mapping approach (9) showed the high quality of the assembled genome, allowing the in-depth comparative genomic analyses. Two close-spaced maca-specific whole genome duplication (WGD) events ∼6.7 million years ago were identified (Figure S6). Multiple genes were identified as potential candidates in the evolution of maca’s decreased leaf surface. Several genes involved in the cold and ultraviolet (UV)-B radiation (Figure S7) were also identified. The *S*-locus genes regulating the self-incompatibility were lost in the maca’s genome. In comparison with *Arabidopsis thaliana* and *Brassica rapa*, thousands of genes under positive selection were identified (6).

Based on the various sequence data, there’s a growing need for integrating these heterogeneous data together. To facilitate both basic biologists and breeders, we set up the Maca Genome Hub (MGH) and integrated the various data including the genome and various annotations, expression data, molecular markers as well as various built-in bioinformatics tools. We believe that MGH will be a valuable resource to boost the rapid development of post-genomics and molecular breeding researches.

## Materials and methods

### Data set

The genome sequence assembly, proteins, general feature format 3 (GFF3) and coding sequences (CDSs) were reported previously and were obtained from the genome sequence consortium. Fragments per kilobase of exon per million fragments mapped for the gene expression was calculated from this study. Chromosomes of *Lepidium* spp. were obtained from the genome count database (5). The non-coding ribonucleic acids (ncRNAs) were predicted relying on CMScan V1.1.2 (http://eddylab.org/infernal/) software against the Rfam V13 database (10). Gene ontology (GO) annotations and predicted pathway information were predicted using InterProScan (5.28–67.0) software and database (11). Simple sequence repeats (SSRs) were mined using MISA software (12) against maca’s genome sequence. We compared the gene similarity between maca and *Arabidopsis* using Basic Local Alignment Search Tool (BLAST) then run the genome-wide gene synteny using MCScanX (13) software with default parameters. The plant transcription factor (TF) gene family database (PlnTFDB) (http://plntfdb.bio.uni-potsdam.de/v3.0/) (14) covers all the families that were templates for characterizing TFs in maca. We employed HMMsearch (15) to identify these TF families, using seeds from Pfam V31 database. If the seeds are not found in the Pfam database, sequences obtained from PlnTFDB were formatted into the seeds using HMMbuild from the toolkit of HMMER (15). All the publications were queried using keyword search `maca’ OR `*Lepidium meyenii*’ in the Pubmed of NCBI (https://www.ncbi.nlm.nih.gov/pmc/) and Google Scholar (http://scholar.google.com). Various introductions to maca were collected from Internet for research purposes only (Figures S8–S11).

### Database development

The MGH database uses Aliyun, one of the largest cloud server providers in the world, thus facilitates MGH outstanding advantages such as (i) scalability in easily expanding its storage size and computing ability, (ii) more stability and (iii) simple to maintain. The MGH relies on the Linux Ubuntu Server 14.04.5, Apache 2.4.7. MGH provides an efficient and friendly interface for users to access a multitude of data, which were displayed on a simple and direct homepage. The search system was created using PHP 5.5.9 and MySQL 5.5.59 software, Html 5, Bootstrap 3.3.7 and jquery 3.2.1, similar to that of our previous database (16).

## Results and discussions

### Comprehensive omics data for maca

Although maca has been a popular herb worldwide, researches on the omics of maca are just at the beginning stage. MGH is aimed to build a comprehensive platform for data storage, data sharing, online analysis, news center and knowledge base for maca research. Currently, besides the genome sequence, MGH has covered the CDSs and predicted proteins, together with full annotations to these genes, including 23 994 GO items, 30 211 Kyoto Encyclopedia of Genes and Genomes (KEGG) pathway items, 96 418 InterPro items and 12 639 Enzyme Commission (EC) items (Table 1). Once a new data set is released on MGH, we will announce it in the news page (Figure S12).

**Table 1:** The data set covered in MGH

**Data set**	**Data source**	**Number**
Genome	Zhang *et al.*, 2016, *Molecular Plant*	641 460
CDS		96 417
Protein		96 417
GFF3		/
KEGG items	This study	30 210
GO items	This study	23 994
Interpro items	This study	96 418
SSR	This study	2461
Publications	PubMed	72
Synteny gene	This study	26 109
Expression Data	This study	12 Data sets
Gene family	This study	5835
TF	This study	54
ncRNA	This study	26 755
miRNA and targets	Sujay Paul, 2017, *3 Biotech*	62/31

The data sources of the genome and annotations come from Zhang *et al*. (6). The miRNA and targets were computationally predicted and experimentally validated by Paul (28).

### Homepage for MGH

**Figure 1:**
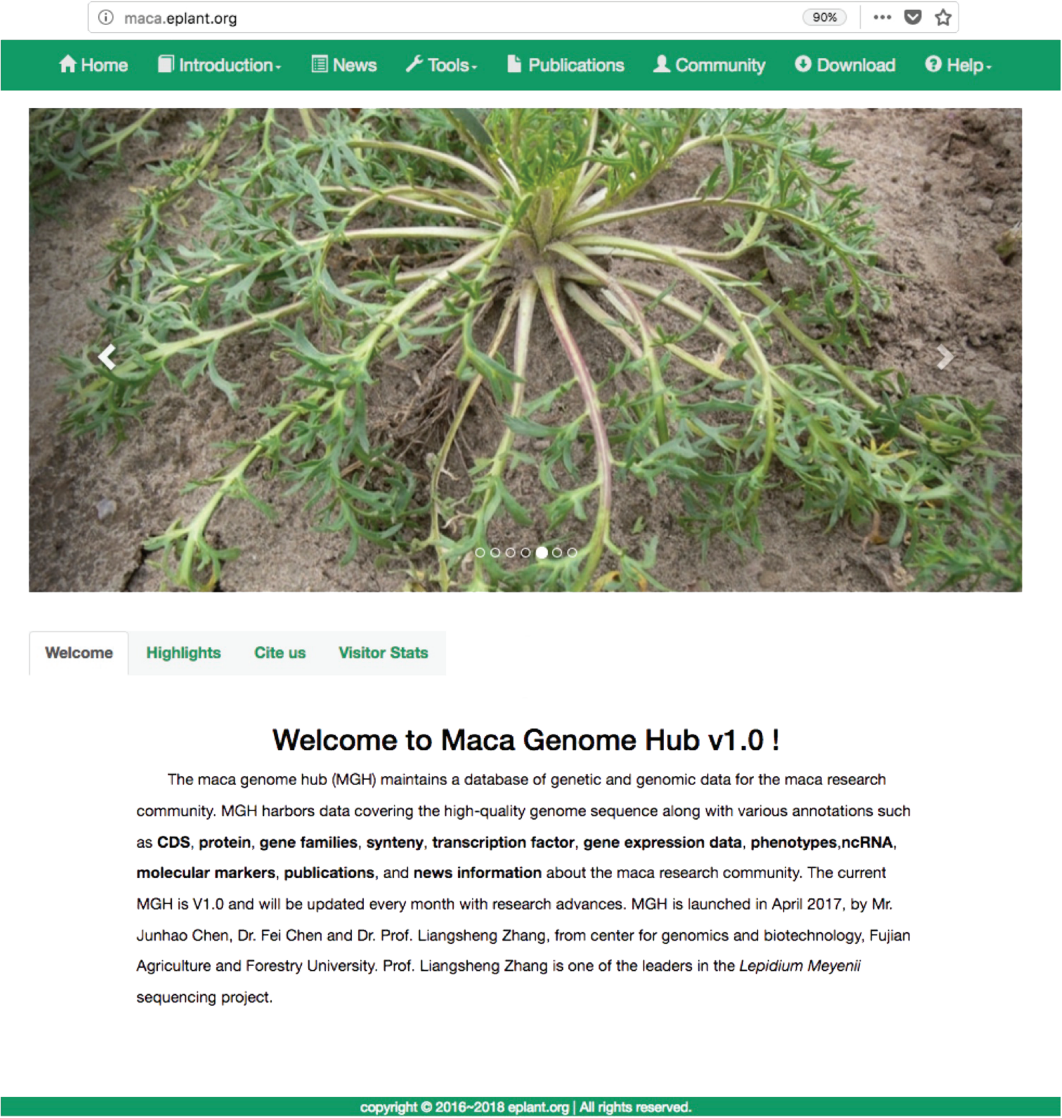
The homepage of MGH. This head part of MGH’s homepage is a menu for quick links of our data, tools, news and help. The sliding pictures show the latest news and representative publications. The database news, highlights, citations, and visitor statistics were shown below the figures.

A user-friendly homepage is essential for attracting worldwide users. We tried our best in designing a homepage for MGH that aims to provide the latest news and updated contents. The current homepage (Figure 1) includes a menu at the head part consisted of eight labels, `Home’, `Introduction’, `News’, `Tools’, `Publication’, `Community’, `Download’ and `Help’. Below the menu are the sliding pictures related to maca research. Pictures now cover the photos of maca, hypocotyl powder, integrated bioinformatics tools and introduction to latest publications. The following part includes four sections, welcome remarks, database highlights, citing information and visitor statistics. The records of visiting access will help us to understand the popularity of MGH database and to upgrade the database.

### Explore the maca genome with BLAST and JBrowse

#### Having a BLAST: searchable and tree-clustered genome resources

**Figure 2:**
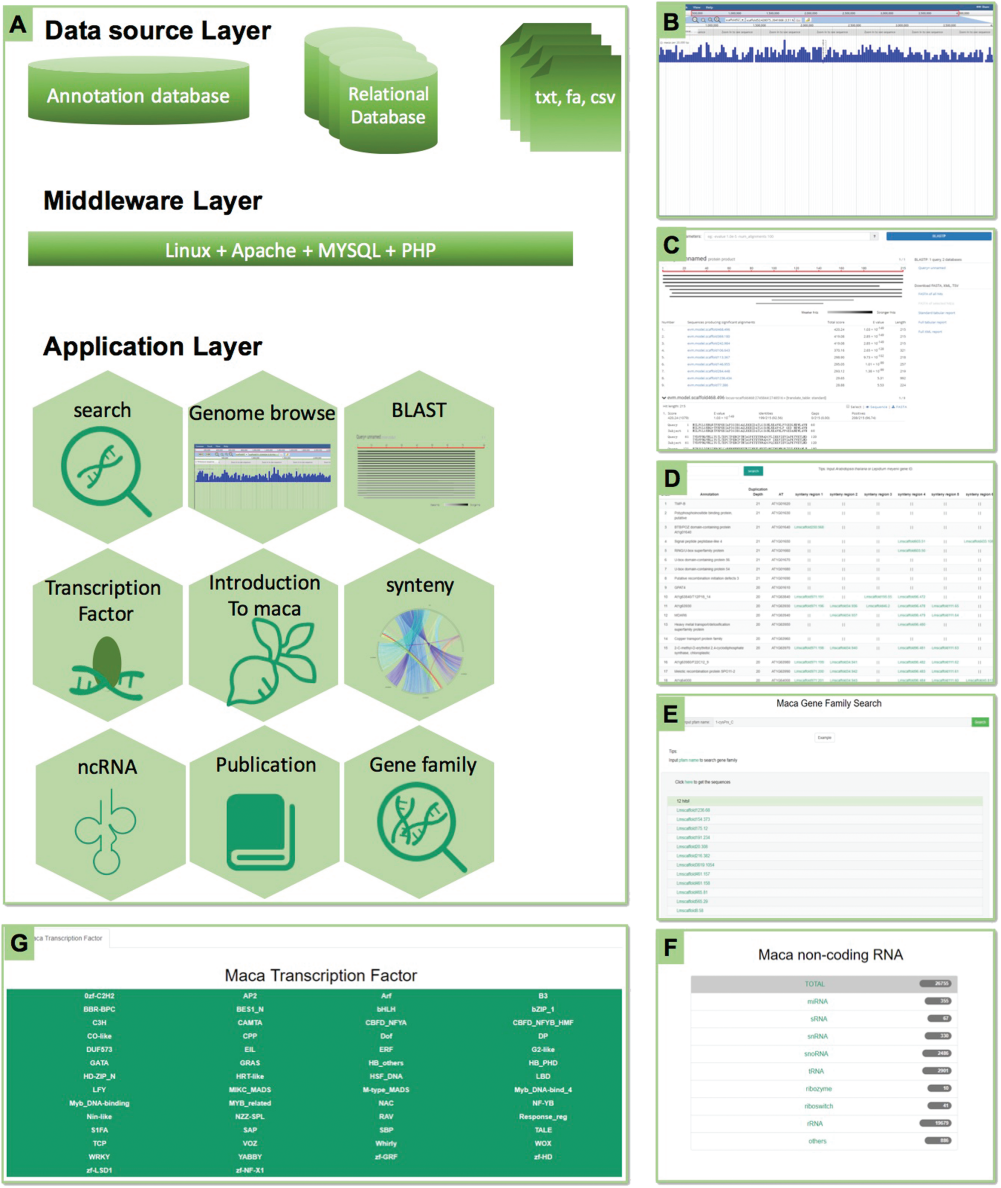
The framework of MGH and some highlights. **(A)** The MGH database consists of three layers: the data source layer stored in the cloud server, the middleware layer for the software and tools and the application layer for multiple data and bioinformatics tools. **(B)** The JBrowse tool for visualizing the genome and genetic sequence. **(C)** The BLAST tool for sequence similarity search. **(D)** The gene synteny between the two Brassicaceae plants *A*. *thaliana* and *L*. *meyenii*. **(E)** Gene family search page. **(F)** The ncRNA page.

BLAST is an extremely popular tool for sequence comparison, and here we adopted the sequence server (17) for online BLAST service. The built-in interface covers the input box that will automatically detect the input sequence and determines whether it is a protein or a DNA sequence (Figure 2). The following part is the parameter box that offers a series of 20 parameters, including eight parameters for general search options, three parameters for query filtering options and nine parameters for restricting the search or results. The following parts include two lists of nucleotide databases (CDS and genome) and a list of protein database. Users can input the sequence in FASTA format, optionally select the parameter and choose the corresponding database then hit the BLAST button for searching. If the user could not find the corresponding database, just type in the keyword, such as `lepidium’ or `Brassicales’ in the search box, the search system will direct users to the data sets by highlighting the keywords. Users could find the graphical display section and the sequence alignment section. Users could download the alignment or sequence by clicking the hits. Batch download is also available. To help users study the gene evolution and facilitate large-scale comparison, we provided >300 green lineage species, with genome, CDS and protein data (Figure S13). These resources and tools will largely satisfy the various demands of maca-related researches.

#### JBrowse for genome visualization

We embedded the JBrowse (V1.11.5) (18) into MGH. Currently, it supports GFF3 annotations and the maca genome sequence. Users could zoom in or zoom out to various scales of any region of interest to find a whole scaffold, a contig or a gene. Users could click the gene and found the details of that gene, including the sequence, intron–exon structure, position and gene length (Figure S14).

### Comparative genetics

#### Gene search

**Figure 3:**
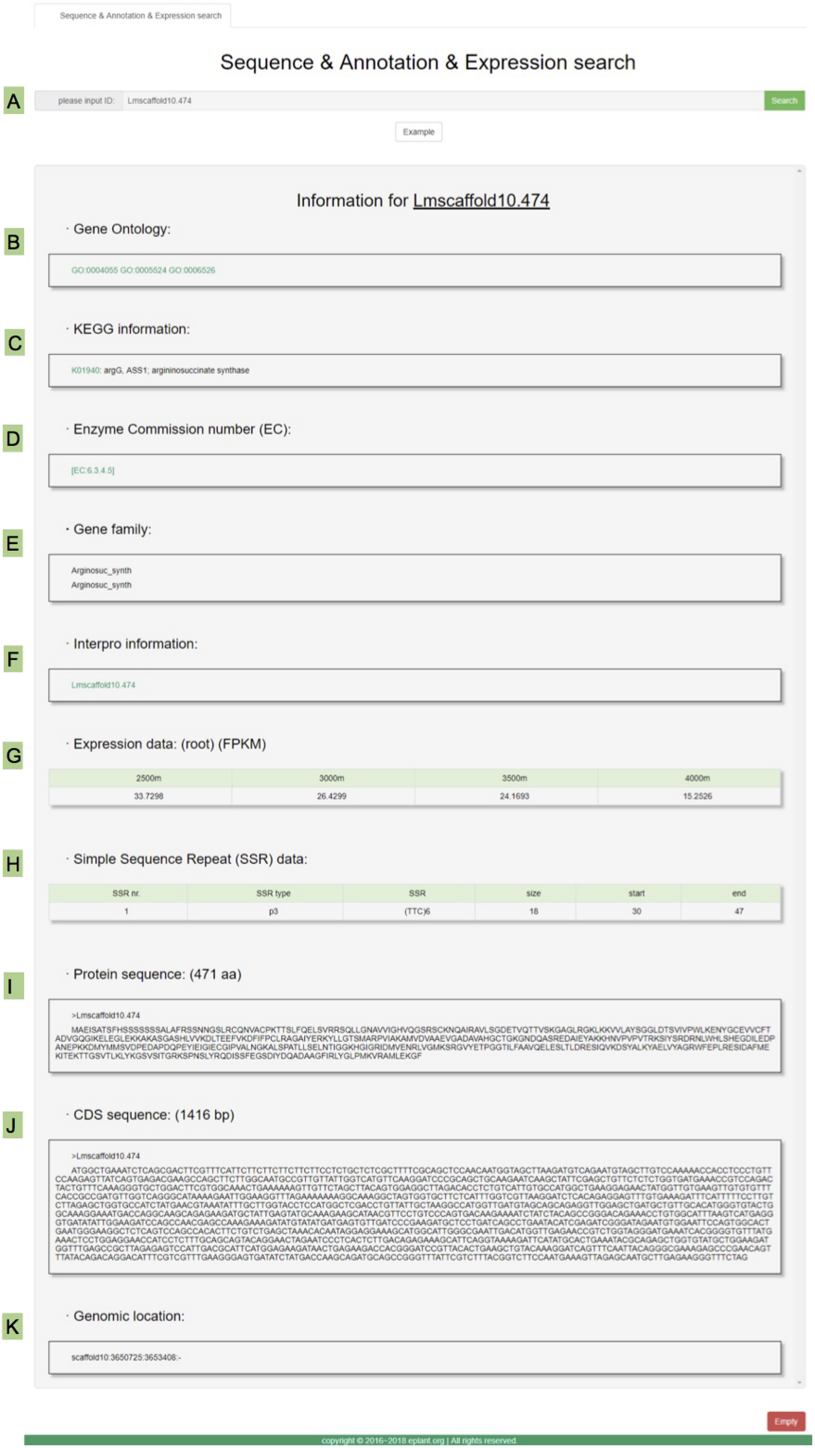
Various information for the genes as shown in the gene search page in MGH. **(A)** The input box and search button. **(B)** The gene ontology annotation. **(C)** The KEGG pathway and related links. **(D)** The EC annotation. **(E)** Gene family information. **(F)** Interpro information for annotations. **(G)** The expression data from four altitude sites. **(H)** SSR marker data. **(I)** Protein sequence. **(J)** CDS sequence. **(K)** The genomic location data.

A search system is perhaps the most popular tool for users to query the gene sequence and related annotations. In MGH, we implemented 96 417 protein-coding genes in the search system. Users simply input the keyword in the input box and click the search button (Figure 3A) then the result will be shown in the return box with a list of gene annotations (Figure 3). These annotations include the GO items (Figure 3B), KEGG items (Figure 3C), EC number (Figure 3D), gene family classifications (Figure 3E), InterPro information (Figure 3F), expression values (Figure 3G), SSR markers (Figure 3H), protein sequence (Figure 3I), coding sequence (Figure 3J) and genomic location (Figure 3K). Link-out information was provided and highlighted in green color.

#### Gene synteny

As a Brassicaceae family species, comparative genomic analysis of maca especially the comparison with the model plant *A*. *thaliana* is an outstanding advantage. To facilitate the easy access to the chromosome level data, we provided the whole genome-wide synteny data of maca and *Arabidopsis*. MGH now offers a search system that allows users to search for genes from either maca or *Arabidopsis*. The search result will direct users to the target gene page. To help users understand the functions of each gene, we provided the brief annotations on the left panel of the webpage. Besides, users could click the target gene to transfer to the gene page for more annotations and to get the sequence (Figure S15).

#### Gene family

A total of 5835 gene families were identified from the 96 417 protein-coding genes. MGH currently offers a search system for users, allowing a quick search of any gene family. Users just need to put in the gene family name as the query and click the search button. The gene family name could be found in the Pfam name link or you can try an example by clicking the example button. The results display a list of members from the gene family. By clicking the name, users will find the gene annotations and sequences. Besides, users are also able to batch download the sequences of the queried gene family (Figure S16).

#### Non-coding RNAs

##### Transcription factors.

TFs such as the well-known WRKY (19, 20) and basic leucine zipper (bZIP) (21, 22) are proteins that control the transcription of a series of genes, thus are popular genes in both basic biology and applied breeding programs (23–26). The defining feature of TFs is that they contain at least one conserved DNA-binding domain that could bind to a specific corresponding DNA sequence, usually in the promoter region. MGH currently hosts 54 TF families. Users could click the name of a TF and found the list of family members. By clicking the name of each gene, the annotation and sequence could be shown. Batch download of all the sequences is also available (Figure S17).

ncRNAs, including transfer RNAs (tRNAs), ribosomal RNAs (rRNAs) and ribozymes, are key elements in genetic information flow. MicroRNAs (miRNAs) are versatile and important regulators in post-transcription with functions in almost every part of plant growth. MGH now offers 10 kinds of ncRNAs with 26 755 items, including miRNAs (355), small RNA (sRNA; 67), small nuclear RNA (snRNA; 330), small nucleolar RNA (snoRNA; 2486), tRNA (2901), ribozyme (10), riboswitch (41), rRNA (19 679) and other undetermined RNAs (886) (Figure S18). Since miRNAs are generally more attractive for a wide range of users, we list all the miRNAs in MGH (Figure S19), with information about the sequence, length, scaffold location and family categorization. The predicted targets were also shown in MGH (Figure S20). Users could quickly identify a specific miRNA or target gene by our search system that allows the input to be a miRNA name, its sequence or the name of a target gene.

### Maca research community

Unlike model plants, maca is a rather under-represented crop; thereby, research people are rather dispersed and not tightly connected currently. We dedicated to build a maca research community toward an open maca information system as is reported in other crops (24) (27). Currently, MGH allows researchers to easily find other people, along with his or her affiliations and mailing addresses. Eighteen research teams were covered and grouped into three research categories: genetics and genomics, plant physiology and medicine and chemistry. As is shown in Figure S21, most of the researchers are involving in the chemistry of maca powder and its application to human. We have selected 75 publications, and they were listed by the publishing date. Full-article portable document formats were provided for research purposes only. Besides, in order to help researchers quickly find related published papers, a quick search system was provided. The search system allows the keyword to be the author, year or the article title (Figure S22). All these publications and data in MGH could be downloaded freely for research purposes (Figure S23).

### Conclusion and future perspective

Maca is an emerging valuable herb plant in both developing and developed countries because of its secondary metabolites in improving human immune responses. It is also a fantastic crop with high-altitude adaptation and cold stress and UV-radiation stress resistance, showing great potential to improve stress tolerance in other crops. To improve the access of genomic data and bioinformatics tools, as well as the relevant information, we build the MGH to store the genome and transcriptome data and to offer various bioinformatics tools for online analysis. The current version 1.0 of MGH also displays multiple breeding information such as growth, molecular markers and various introductions for breeders.

With the rapid development of sequencing technology and bioinformatics tools, more and more transcriptomes and population genomic data will be sequenced. MGH is dedicated to updating the information and tools whenever new data sets are available and welcome users to submit their data or ideas with us. Besides, MGH is targeted to be an online community to unite worldwide researchers and hope to develop a network for the research community such as the iFacebook in InsectBase (2). Also, if users have any problem or conflict of interest, please feel free to contact the developing team (Figure S24) who are dedicated to helping users at any time. We believe this resource and future updates will be a valuable resource to boost the maca researches.

## Supplementary Material

Supplementary Data

## Notes

**Database URL:** http://maca.eplant.org
