# Supplementary figures and images for: MGH: a genome hub for the medicinal plant maca (Lepidium meyenii)
Source: Database (Oxford). 2018 Oct 19;2018:bay113. doi: 10.1093/database/bay113 (PMC6195103; doi:10.1093/database/bay113)

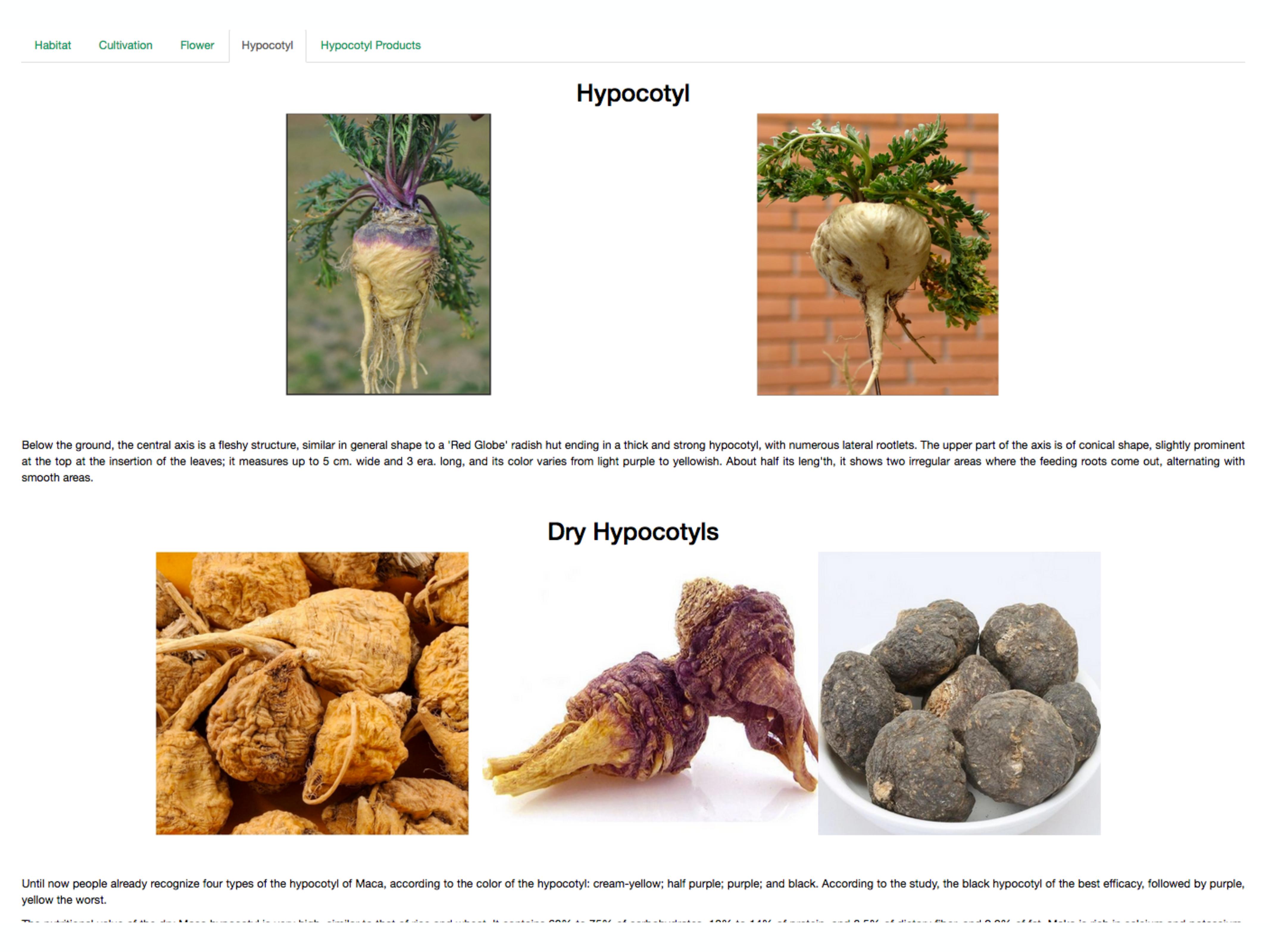

Supplement: Supplementary Data [file bay113_suppl_data.zip › figs10.jpg]

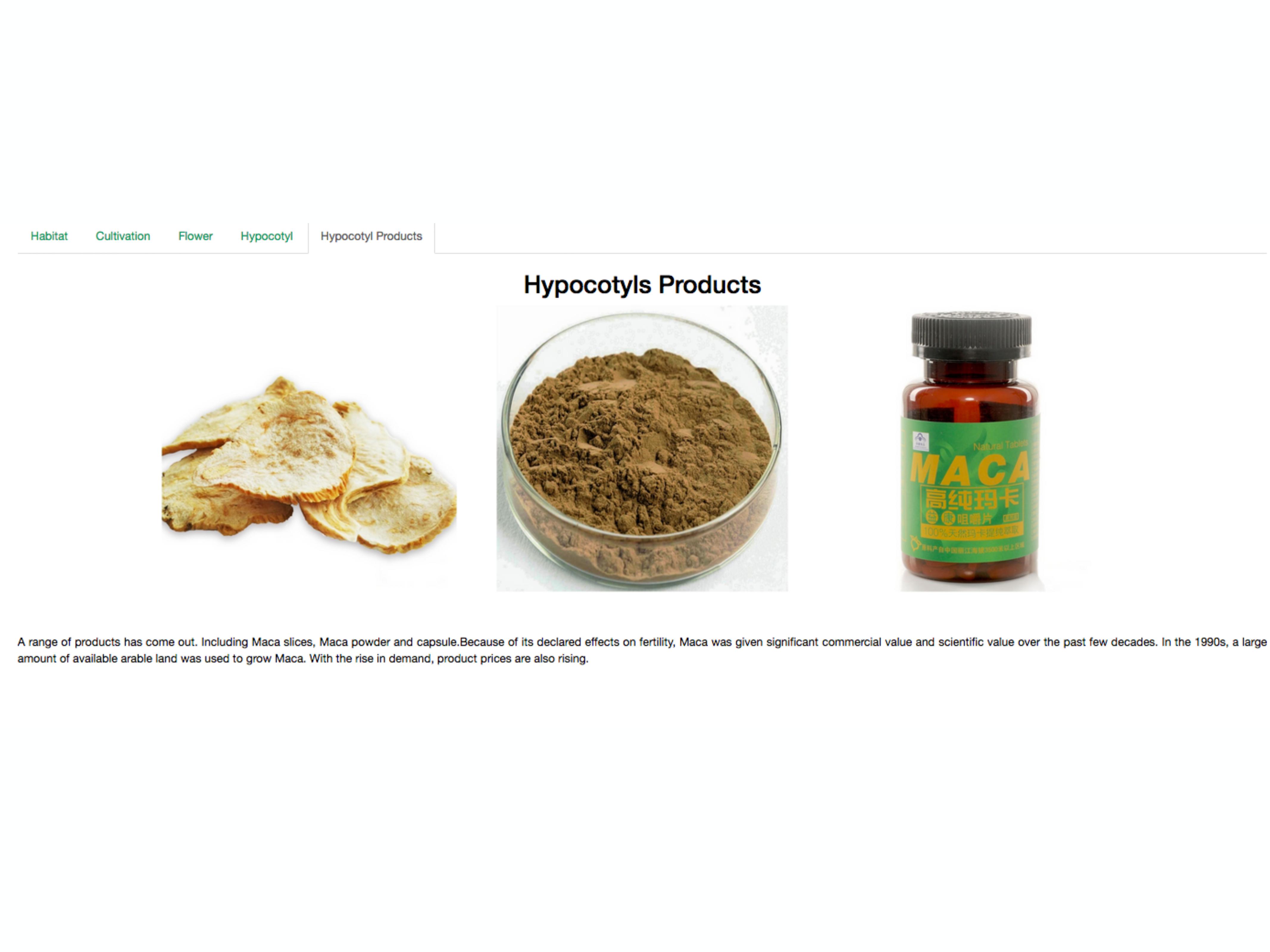

Supplement: Supplementary Data [file bay113_suppl_data.zip › figs11.jpg]

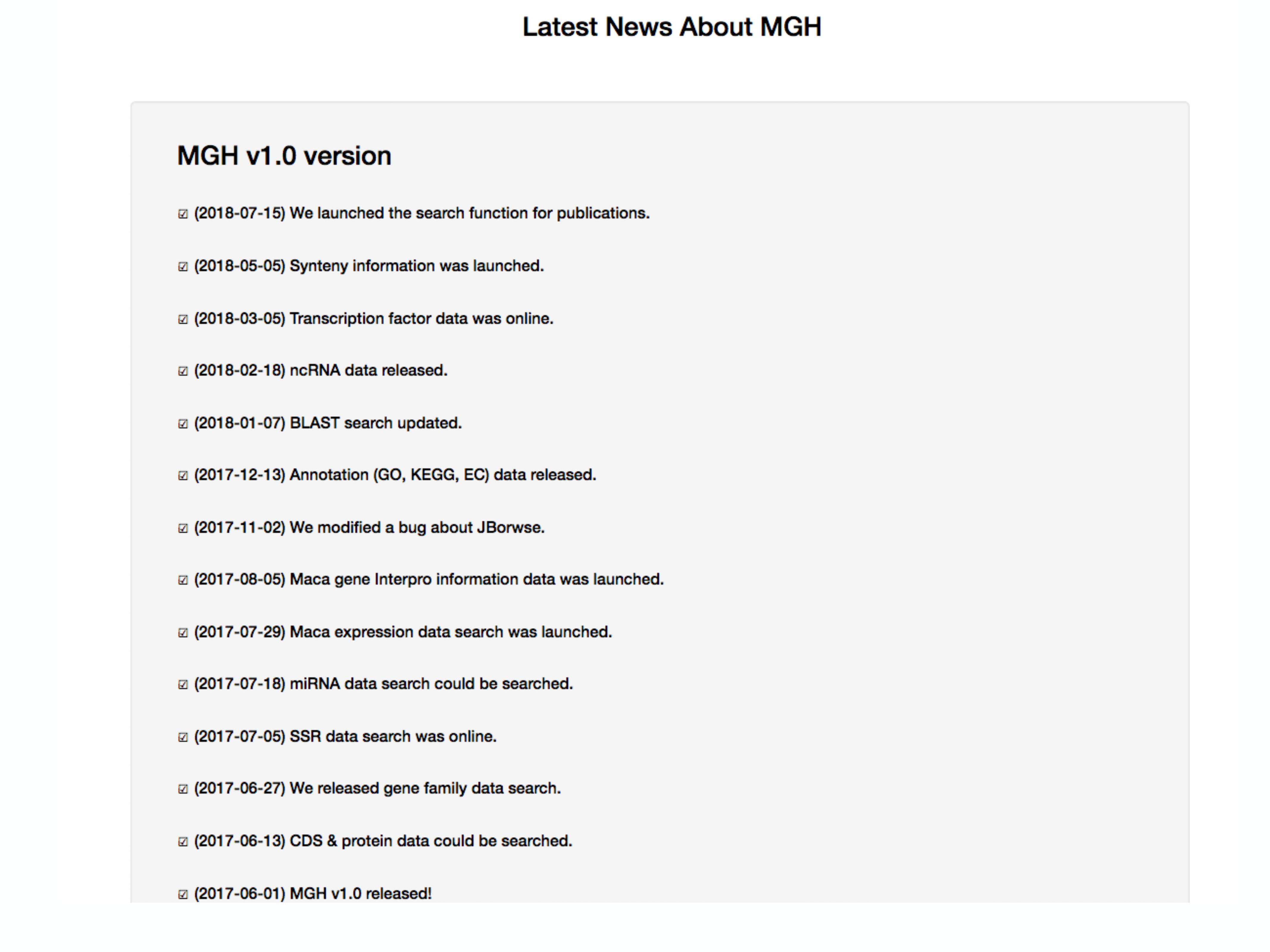

Supplement: Supplementary Data [file bay113_suppl_data.zip › figs12.jpg]

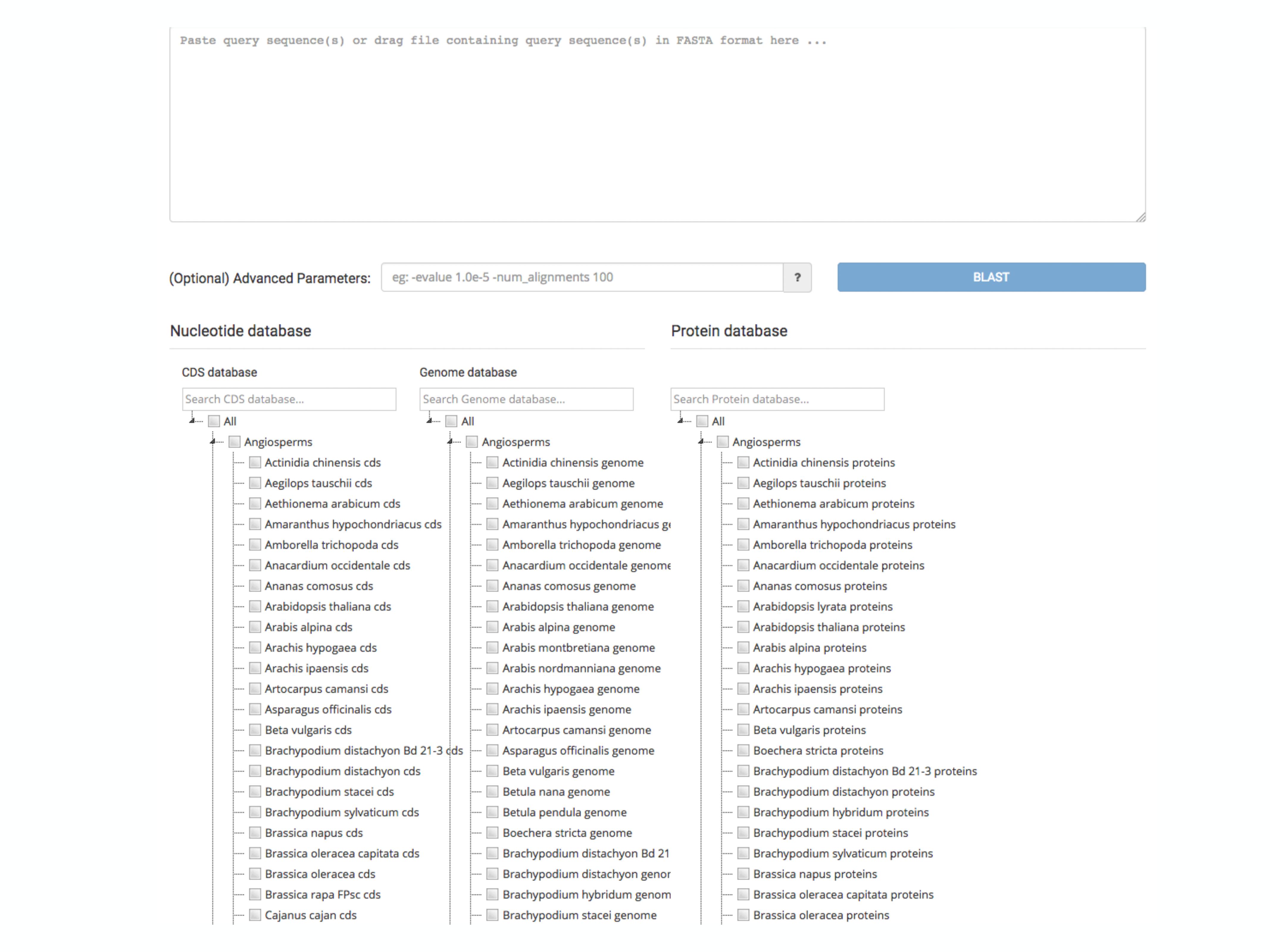

Supplement: Supplementary Data [file bay113_suppl_data.zip › figs13.jpg]

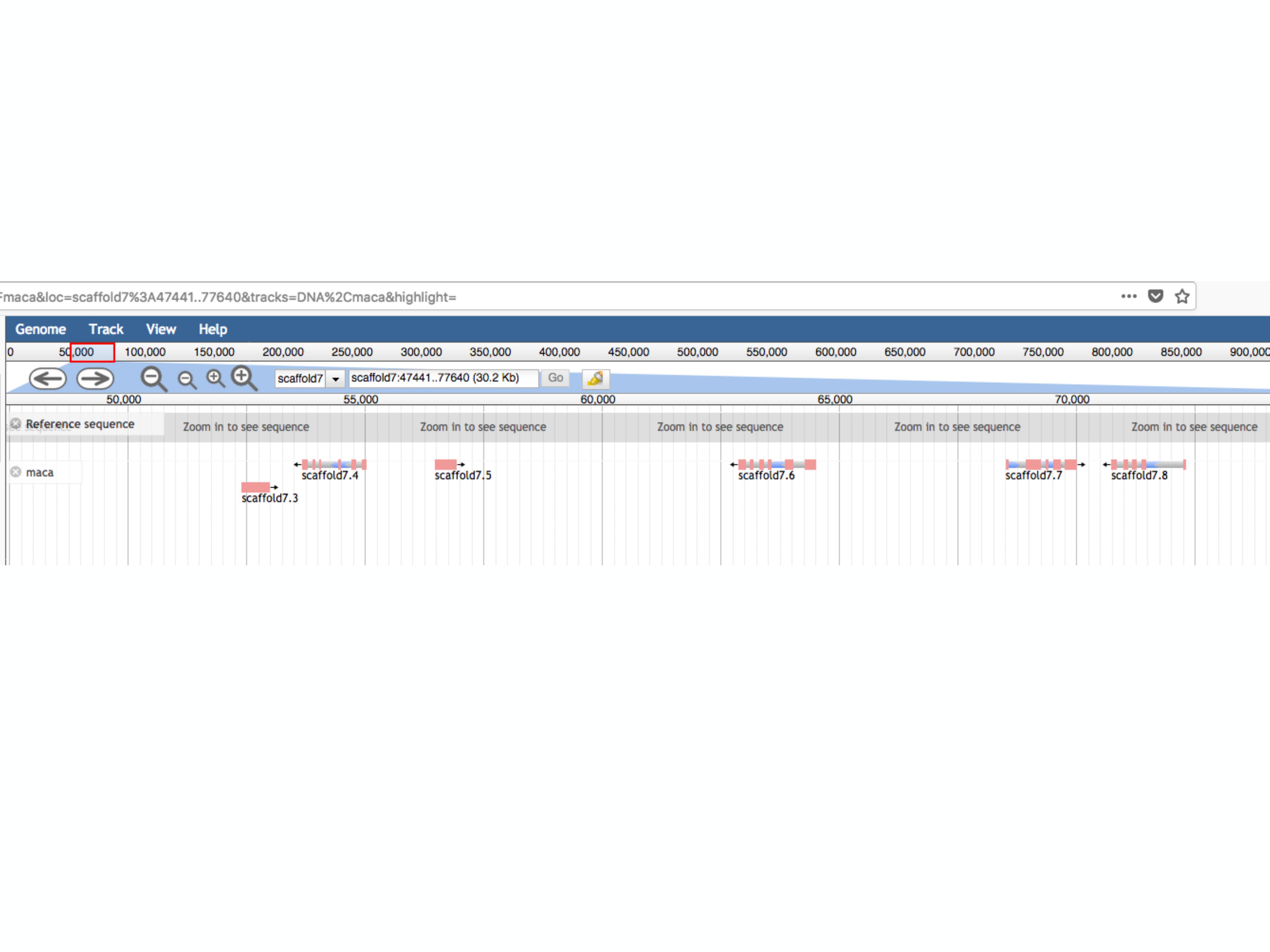

Supplement: Supplementary Data [file bay113_suppl_data.zip › figs14.jpg]

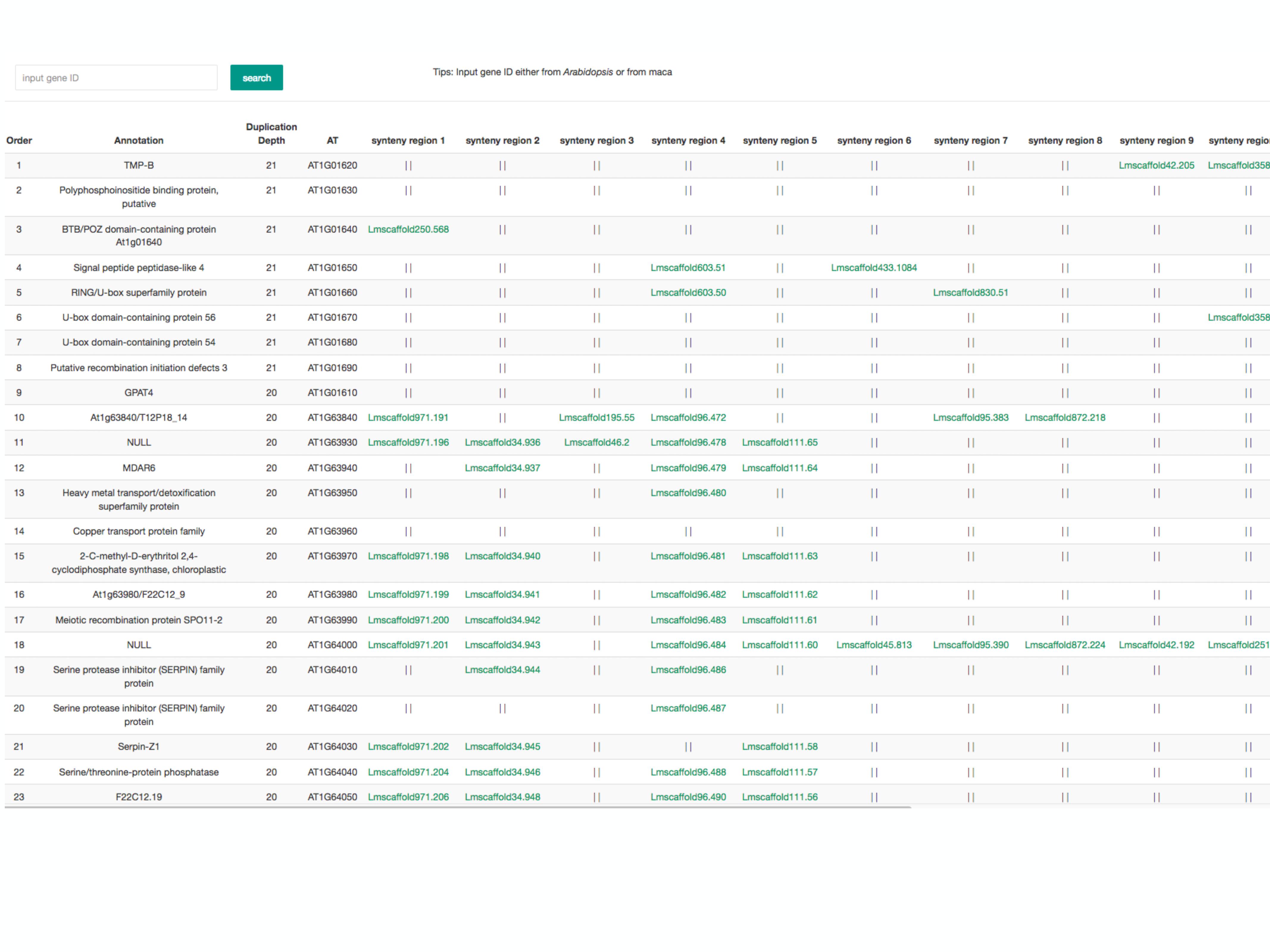

Supplement: Supplementary Data [file bay113_suppl_data.zip › figs15.jpg]

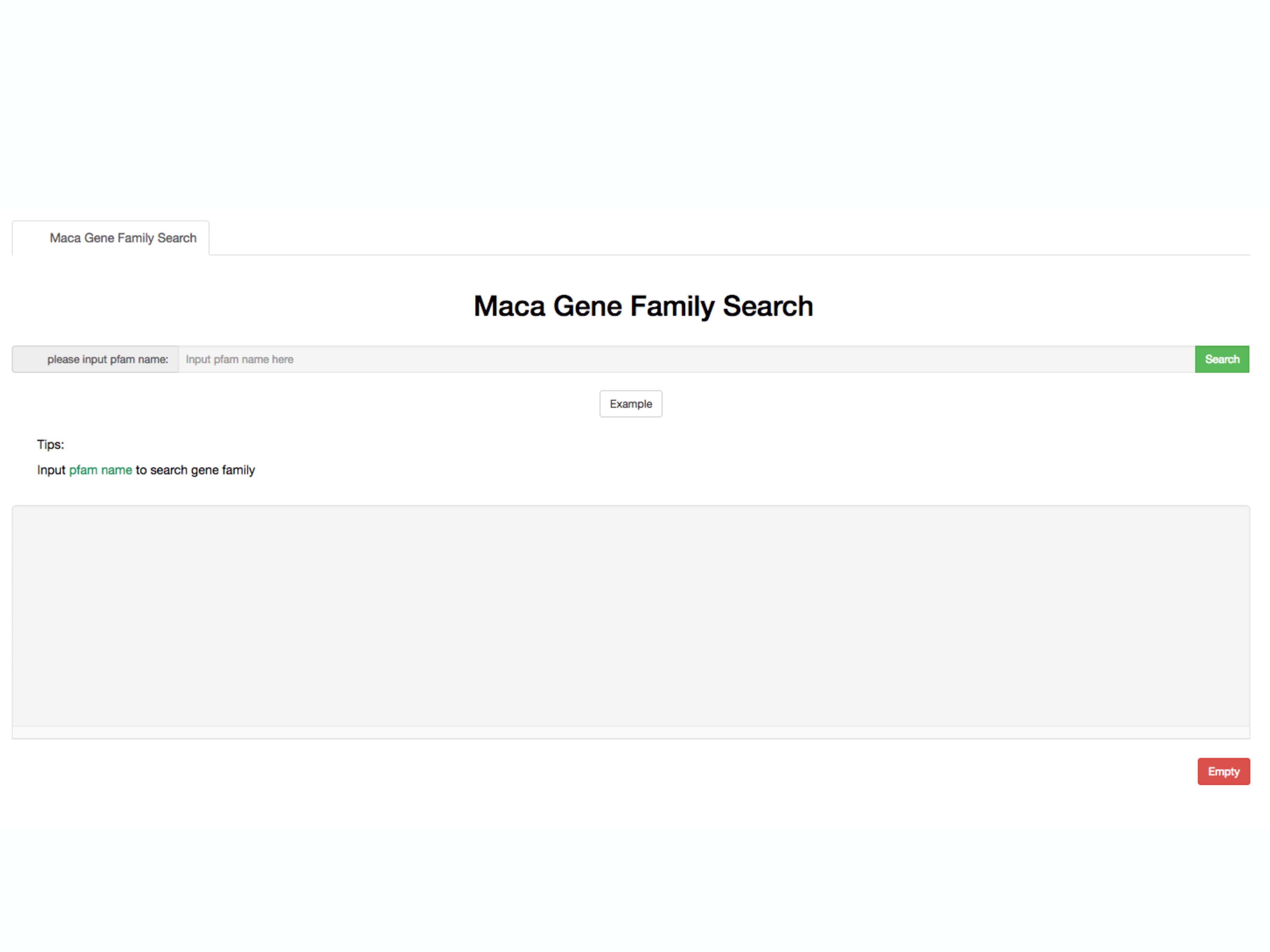

Supplement: Supplementary Data [file bay113_suppl_data.zip › figs16.jpg]

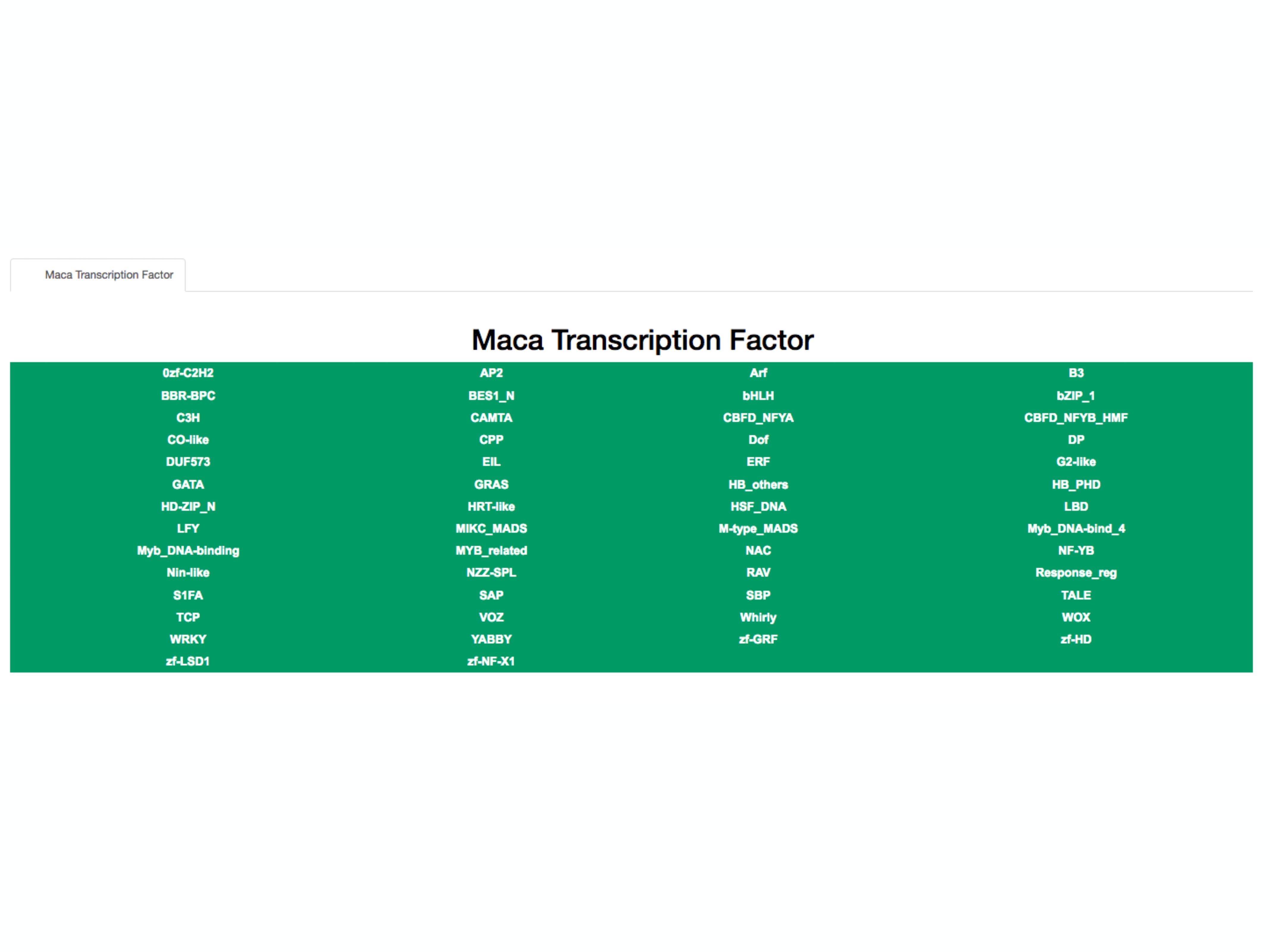

Supplement: Supplementary Data [file bay113_suppl_data.zip › figs17.jpg]

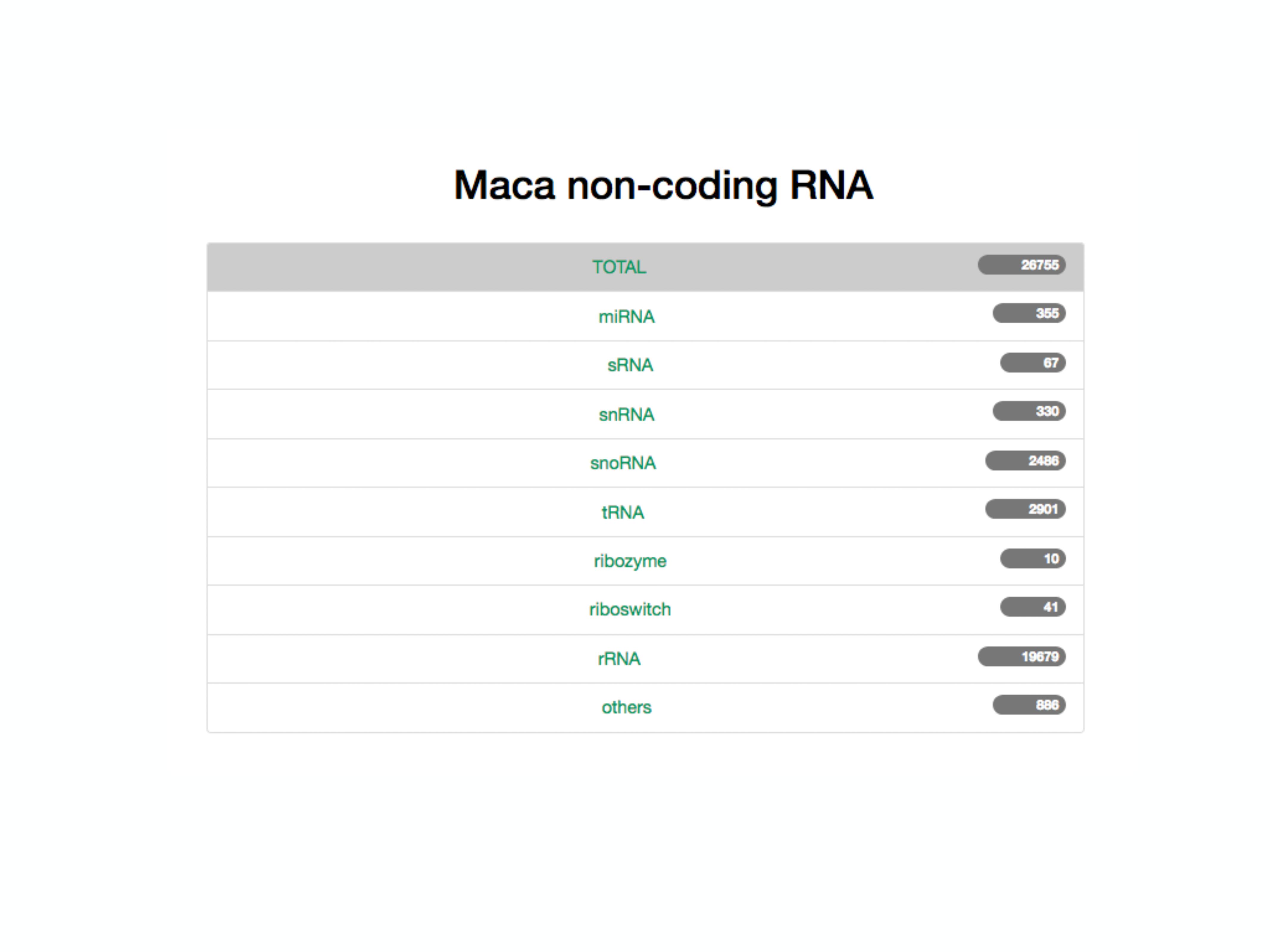

Supplement: Supplementary Data [file bay113_suppl_data.zip › figs18.jpg]

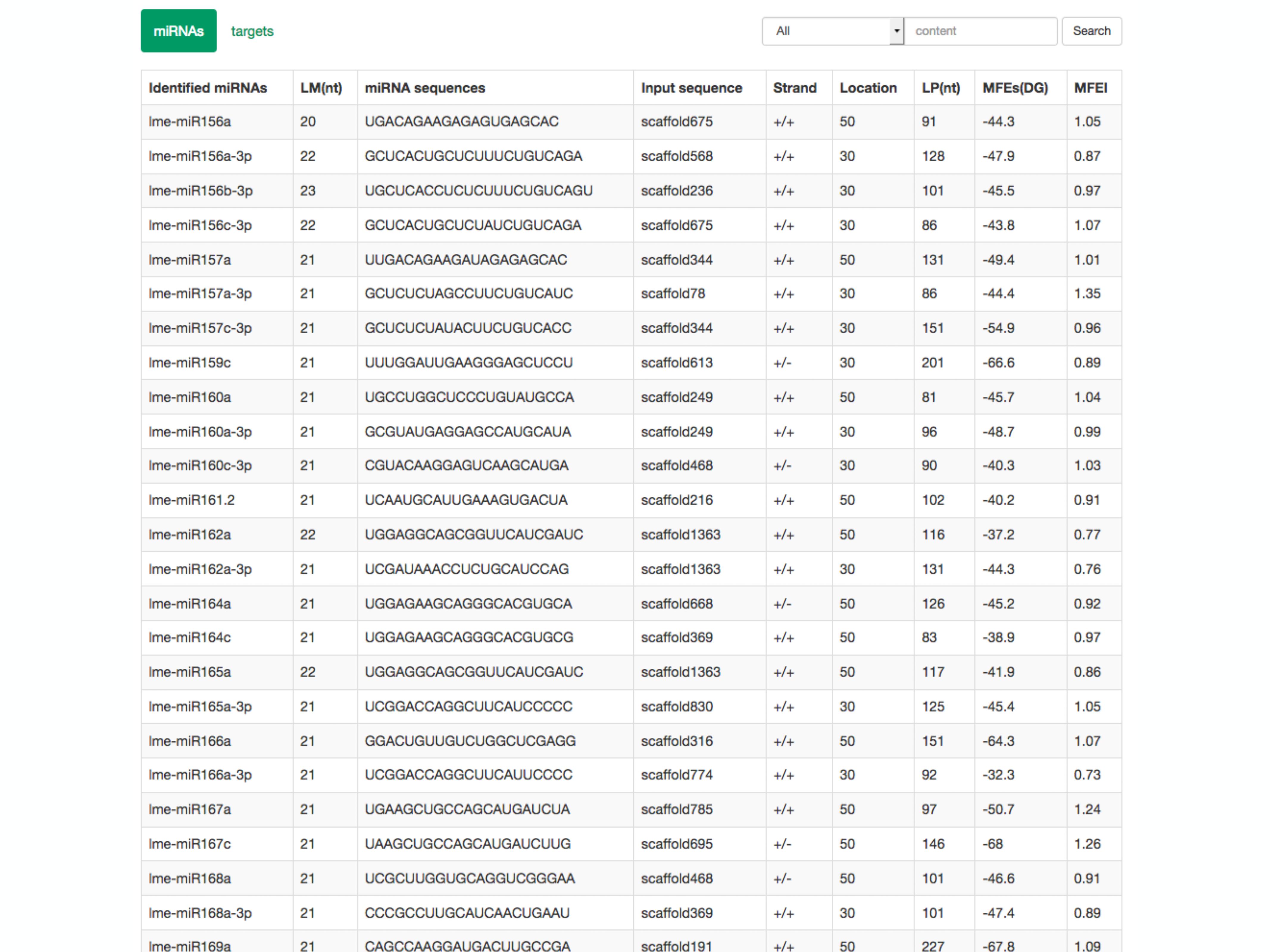

Supplement: Supplementary Data [file bay113_suppl_data.zip › figs19.jpg]

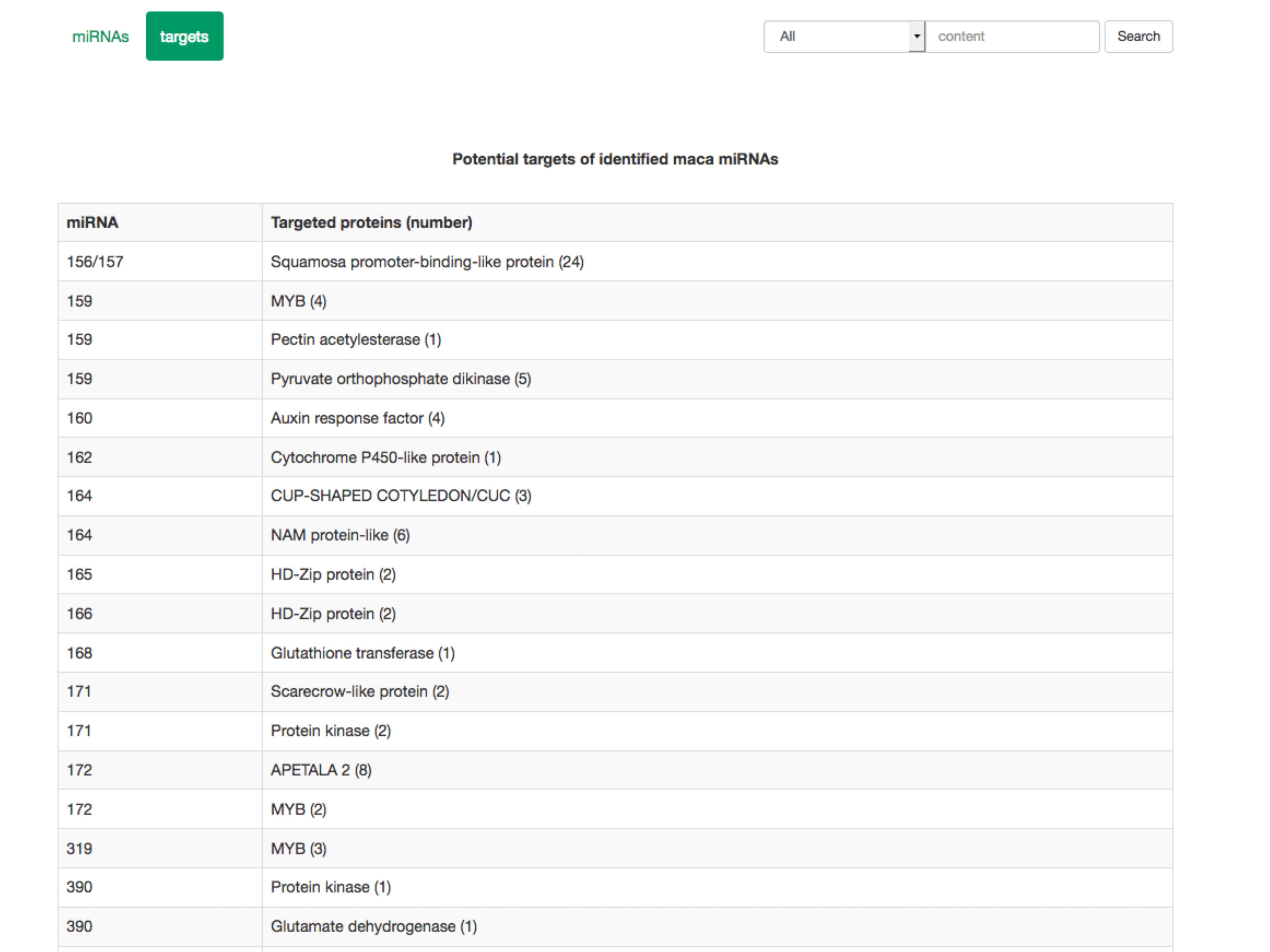

Supplement: Supplementary Data [file bay113_suppl_data.zip › figs20.jpg]

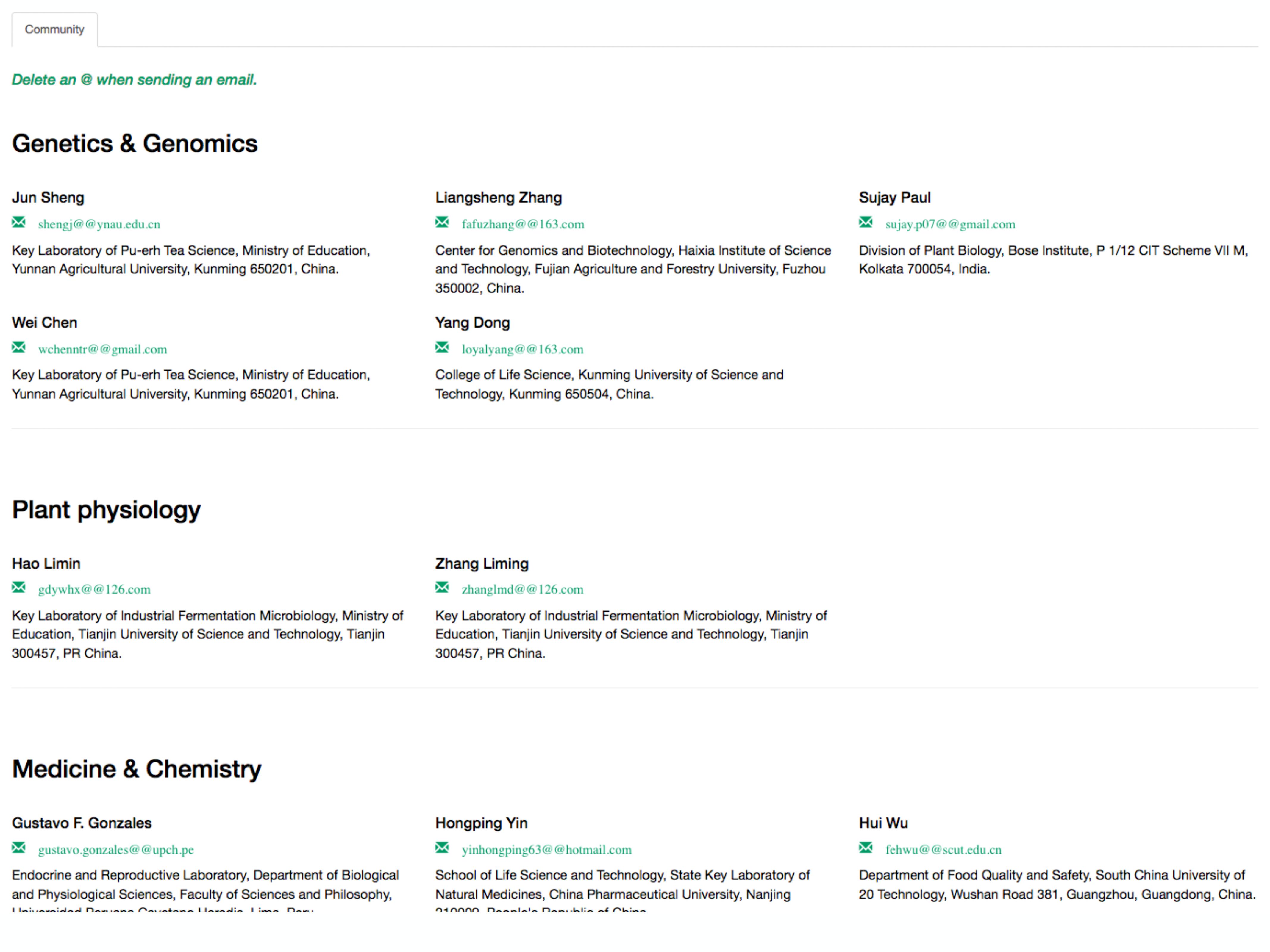

Supplement: Supplementary Data [file bay113_suppl_data.zip › figs21.jpg]

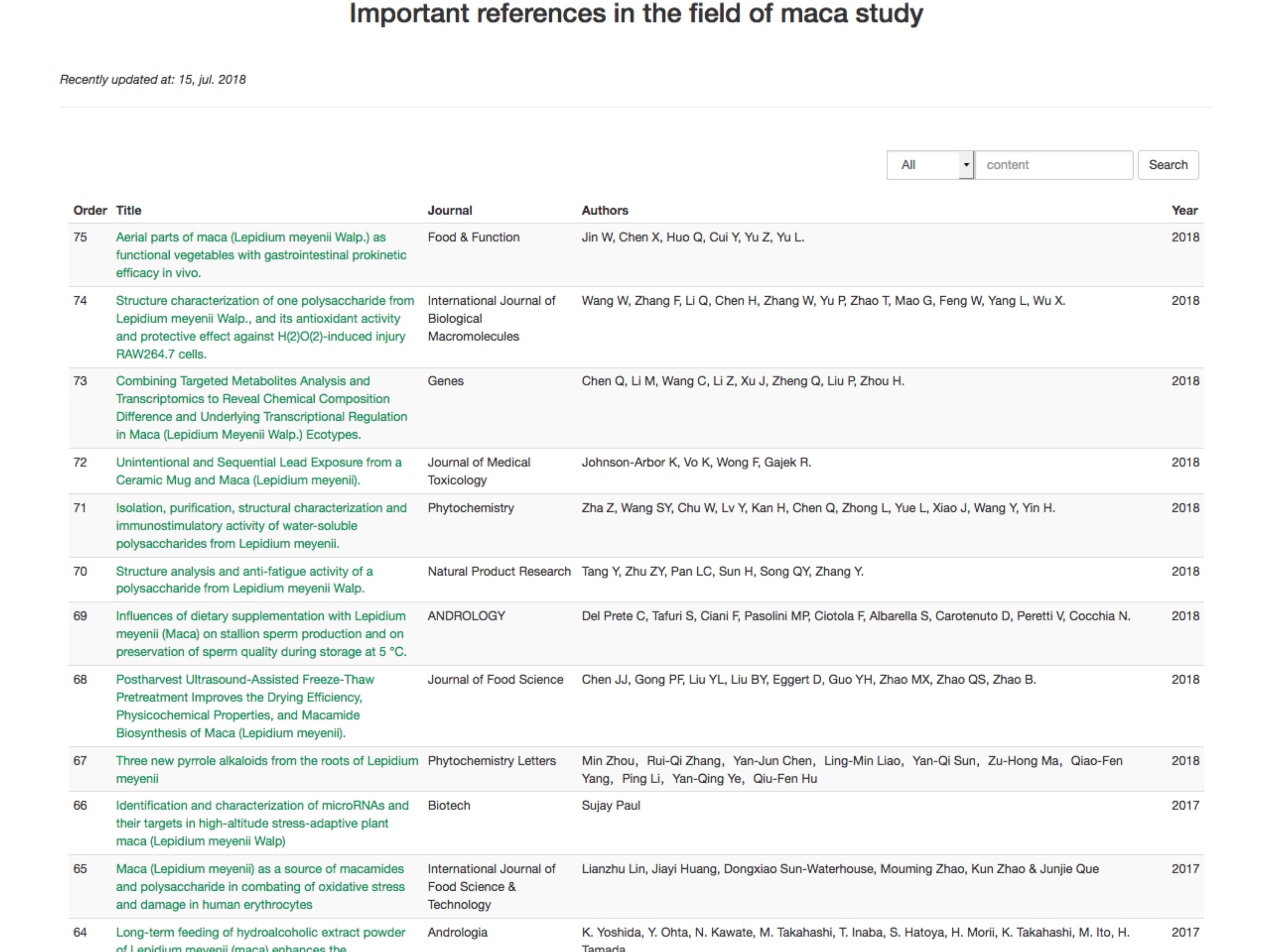

Supplement: Supplementary Data [file bay113_suppl_data.zip › figs22.jpg]

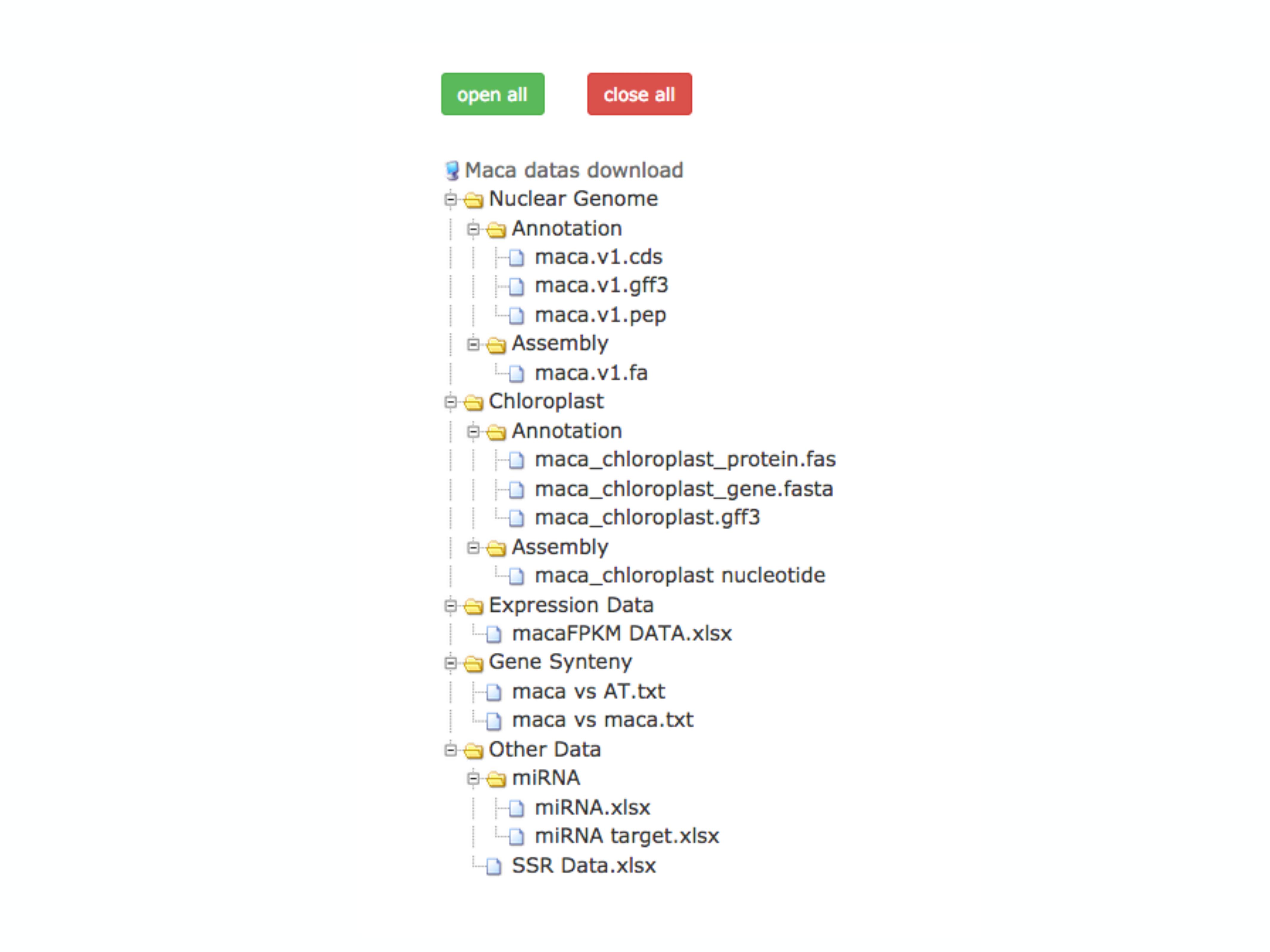

Supplement: Supplementary Data [file bay113_suppl_data.zip › figs23.jpg]

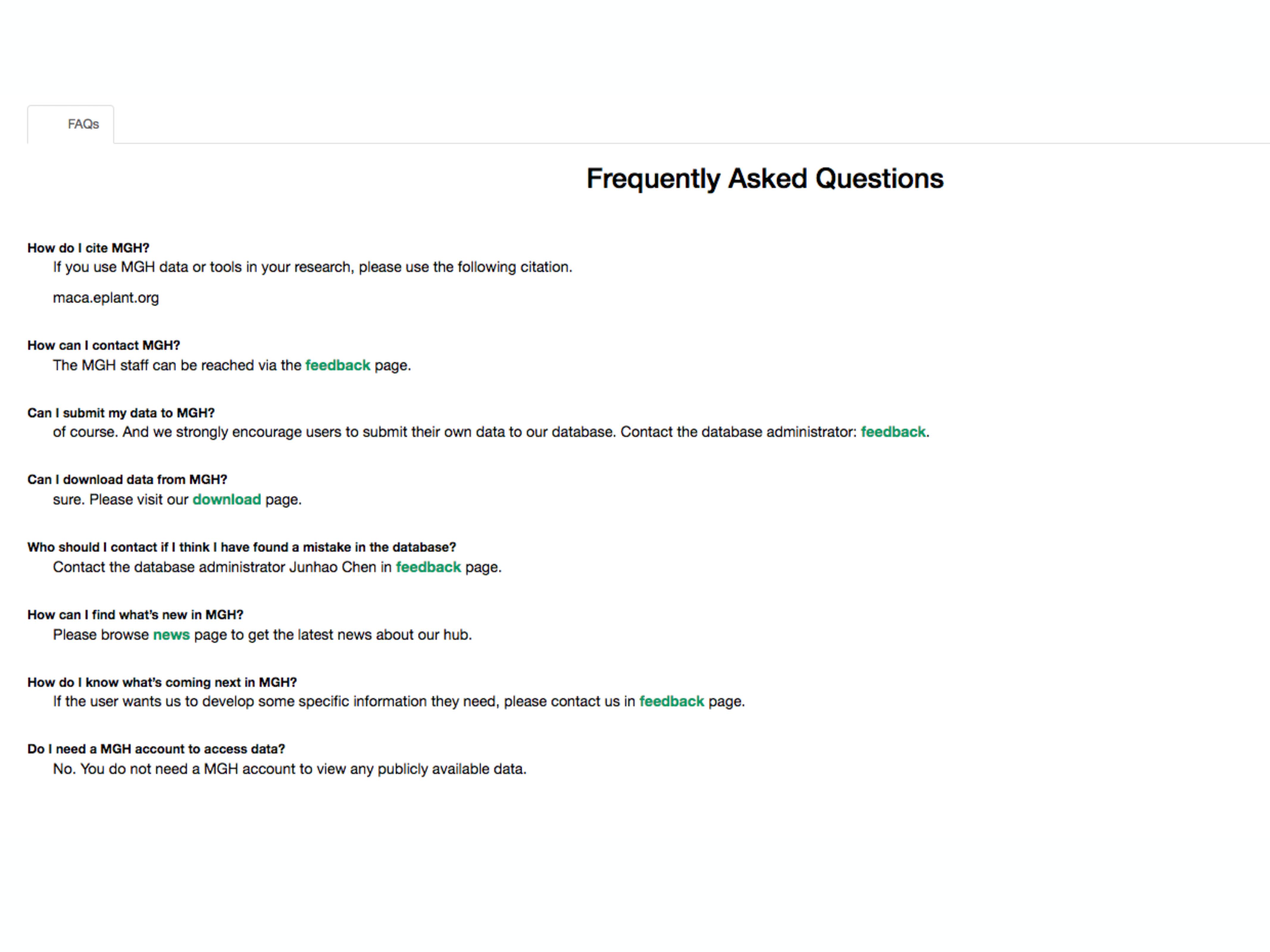

Supplement: Supplementary Data [file bay113_suppl_data.zip › figs24.jpg]

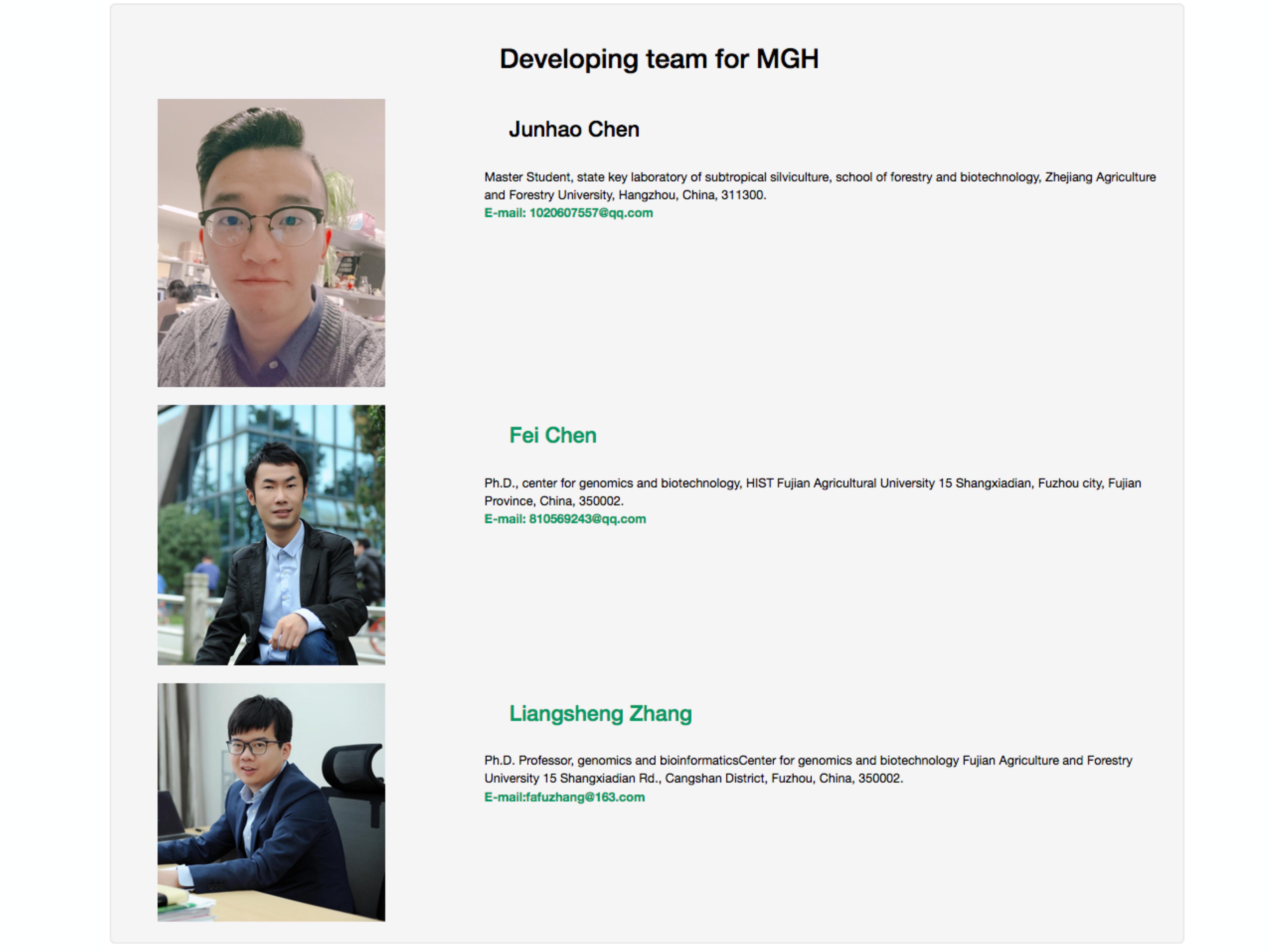

Supplement: Supplementary Data [file bay113_suppl_data.zip › figs25.jpg]

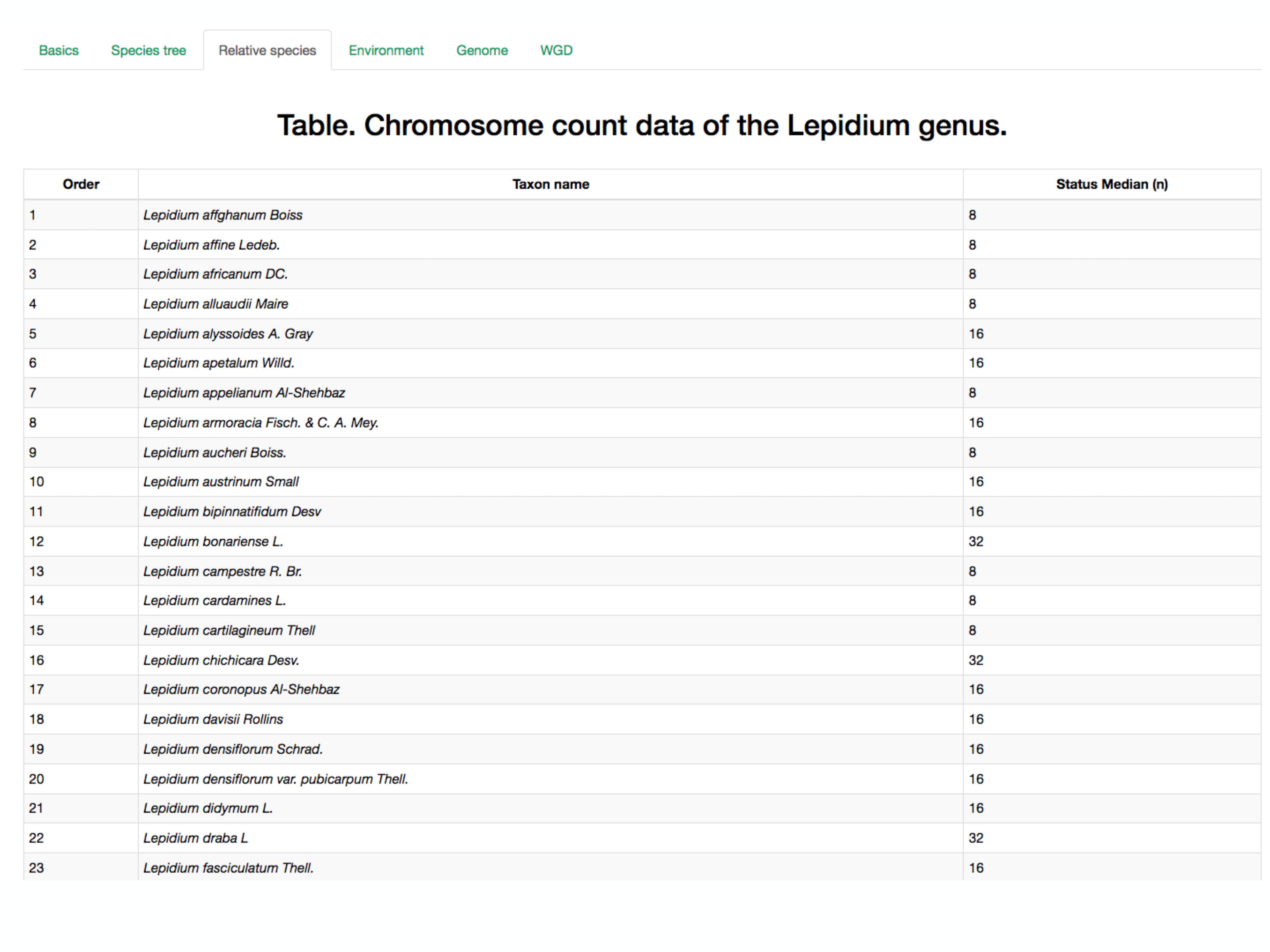

Supplement: Supplementary Data [file bay113_suppl_data.zip › figs3.jpg]

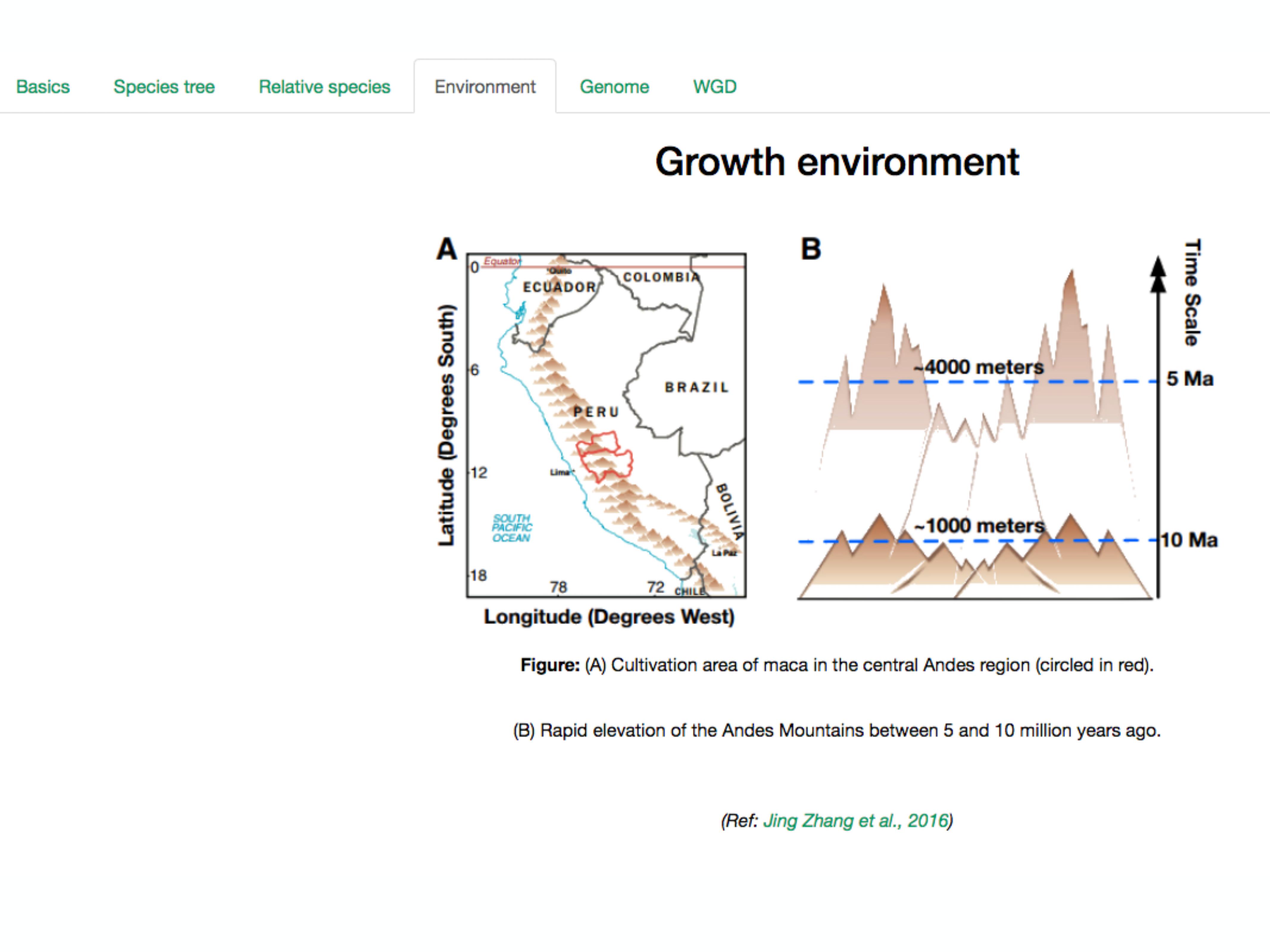

Supplement: Supplementary Data [file bay113_suppl_data.zip › figs4.jpg]

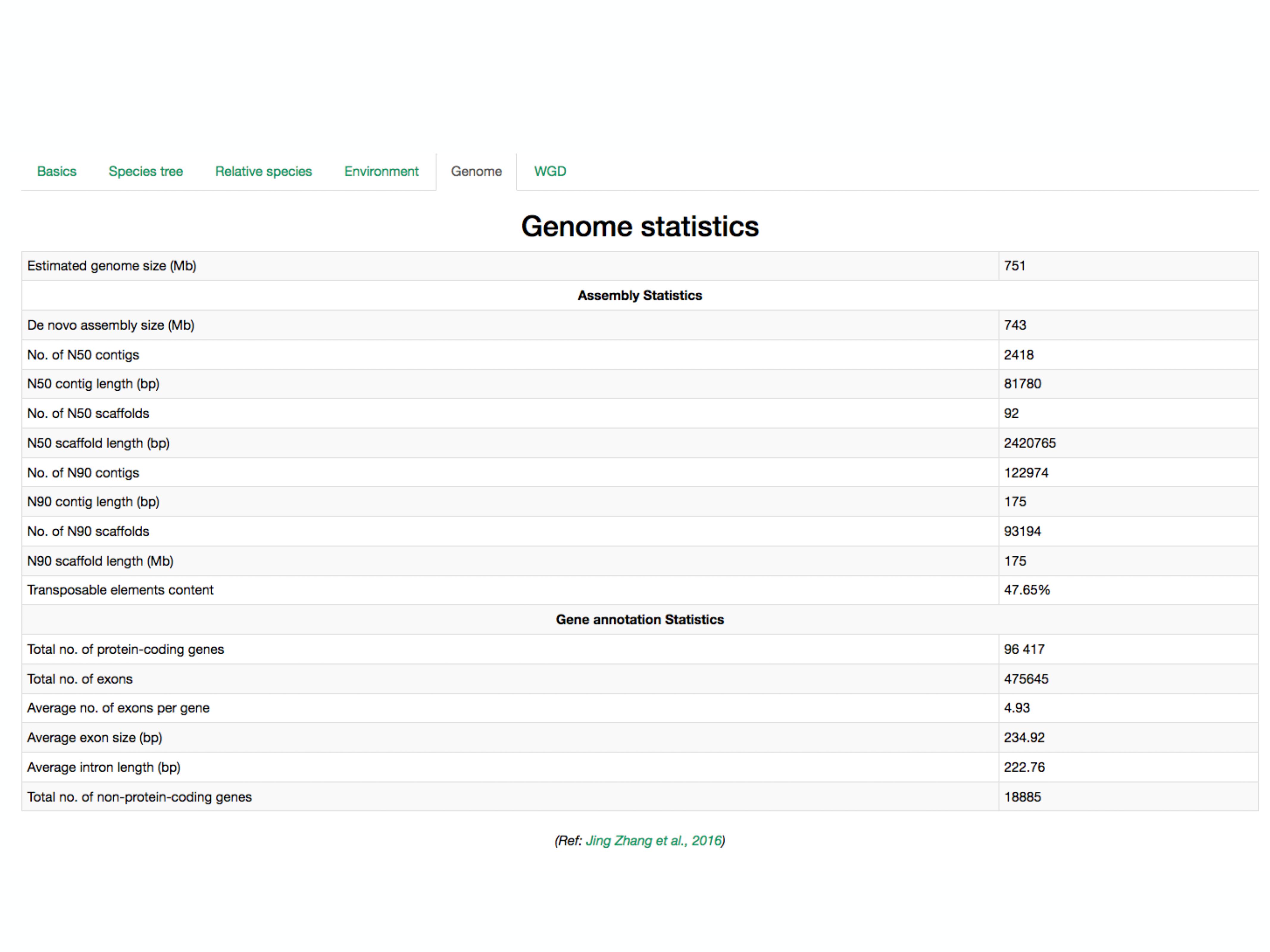

Supplement: Supplementary Data [file bay113_suppl_data.zip › figs5.jpg]

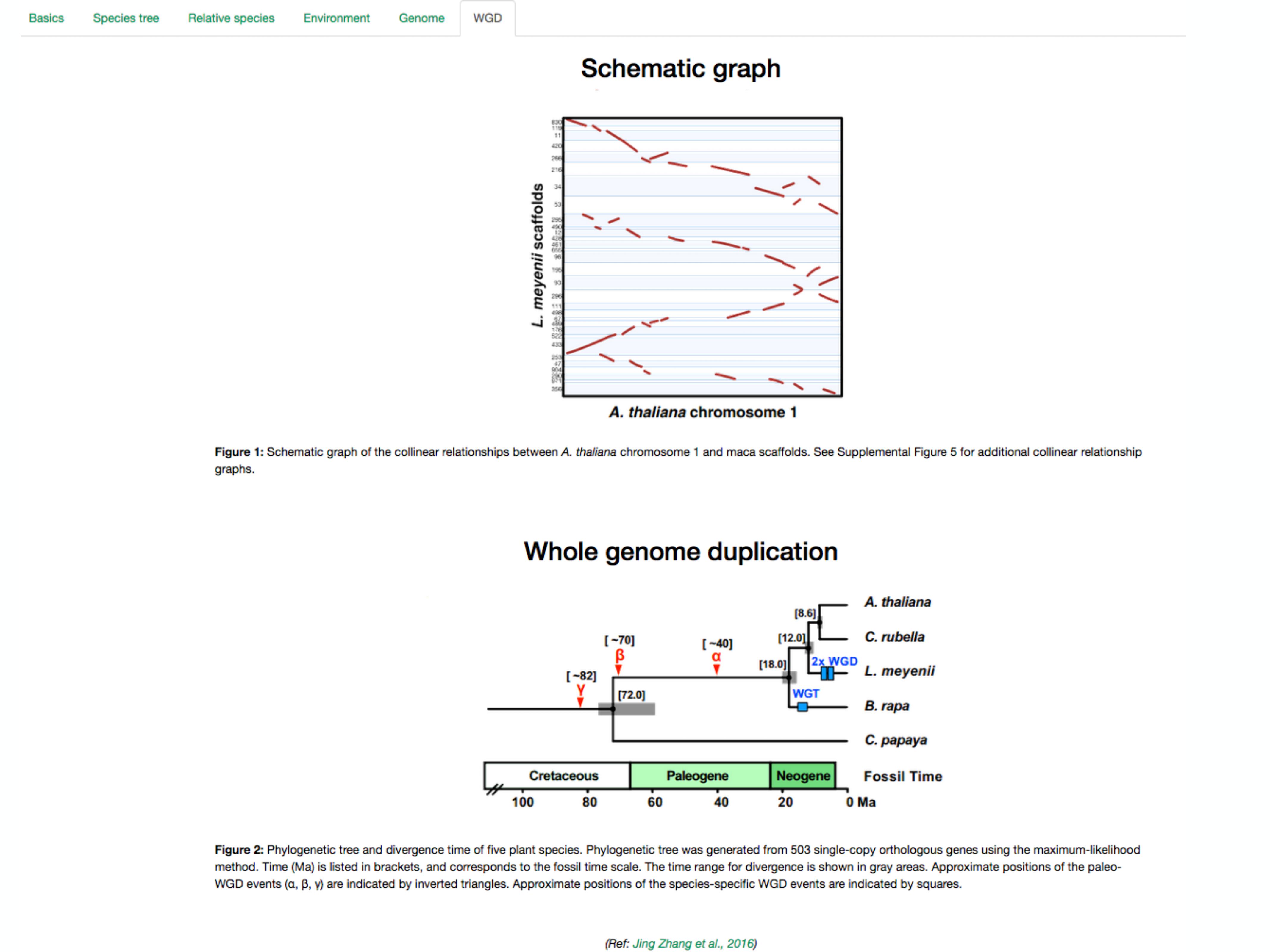

Supplement: Supplementary Data [file bay113_suppl_data.zip › figs6.jpg]

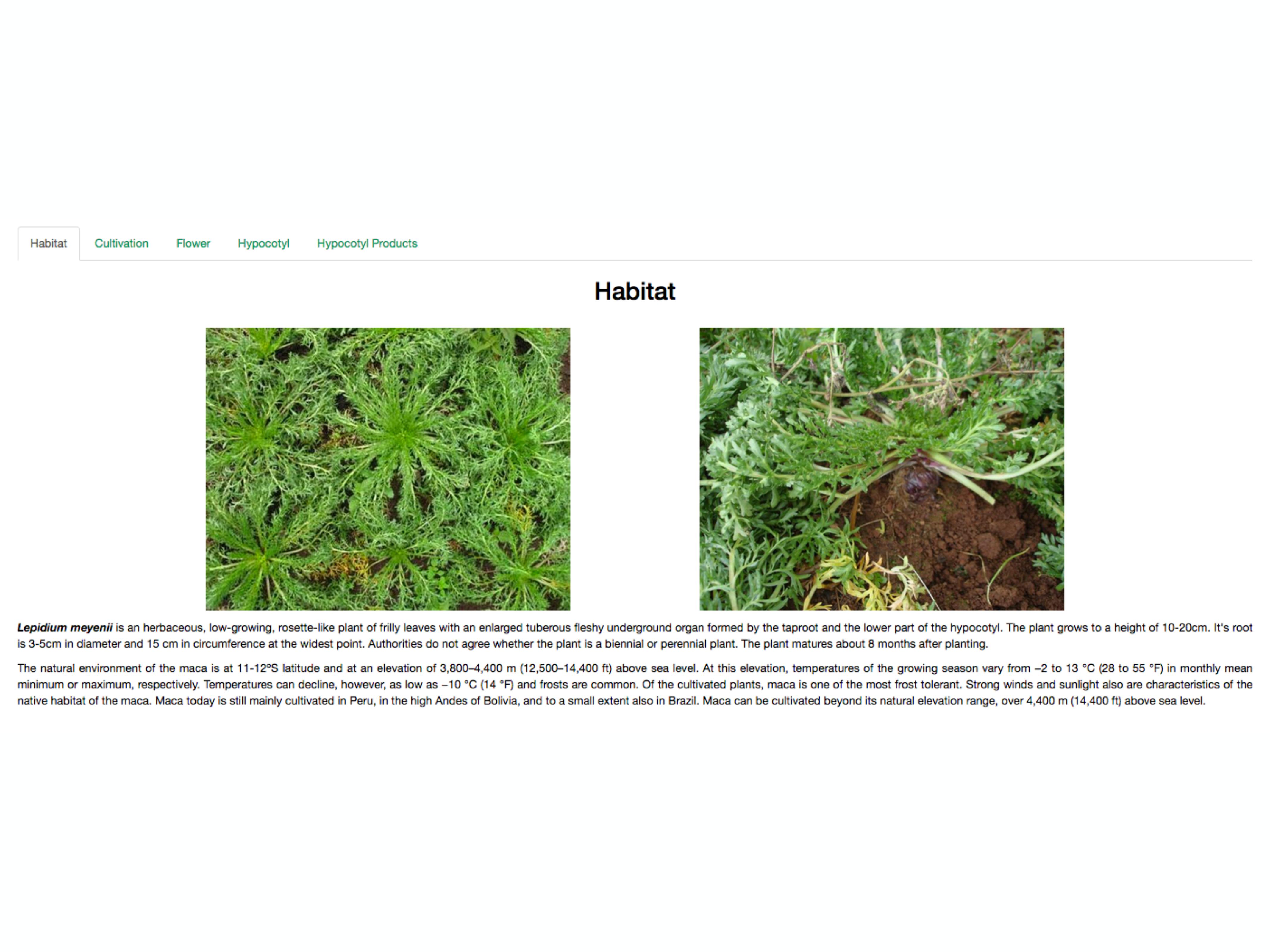

Supplement: Supplementary Data [file bay113_suppl_data.zip › figs7.jpg]

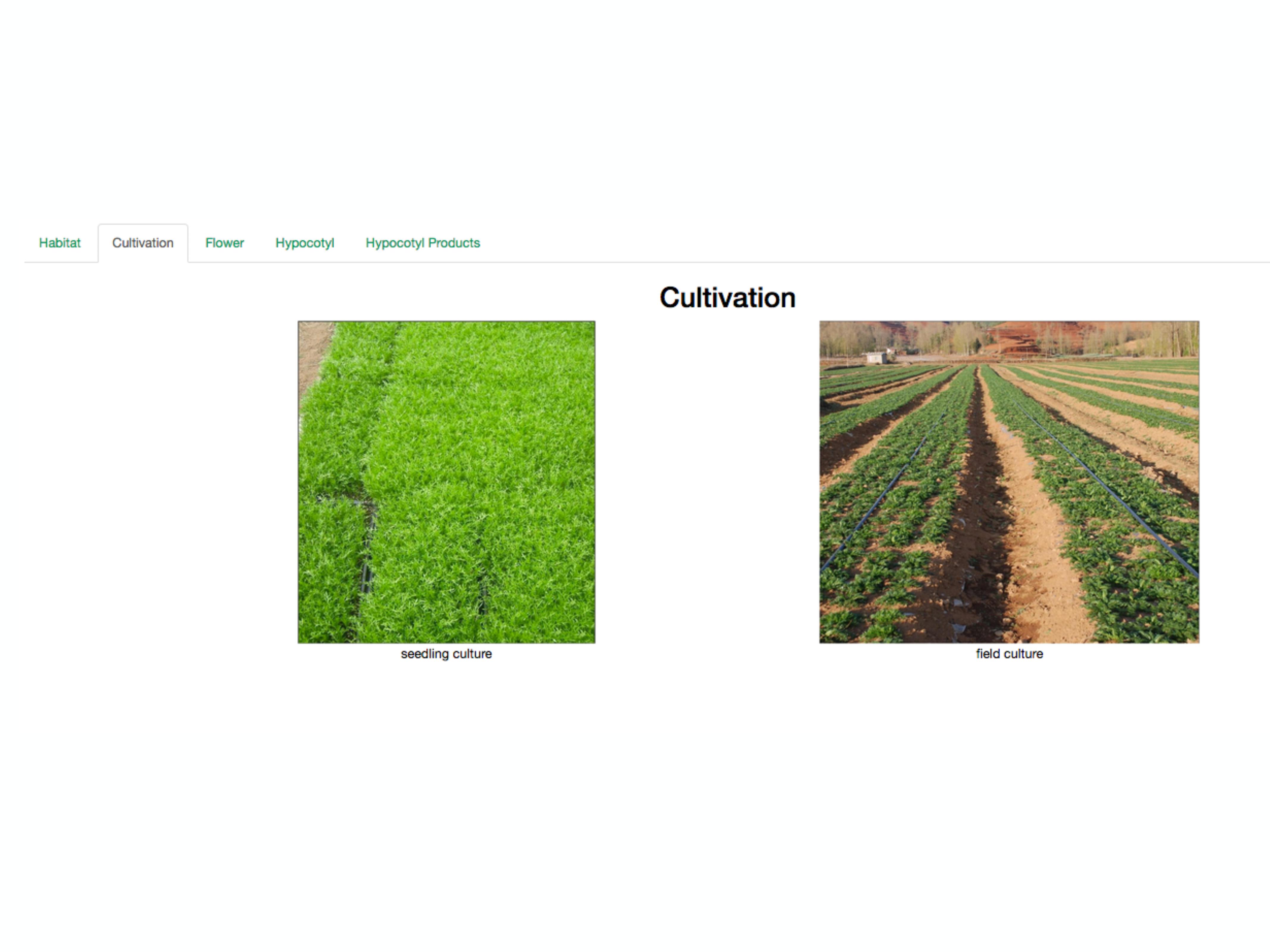

Supplement: Supplementary Data [file bay113_suppl_data.zip › figs8.jpg]

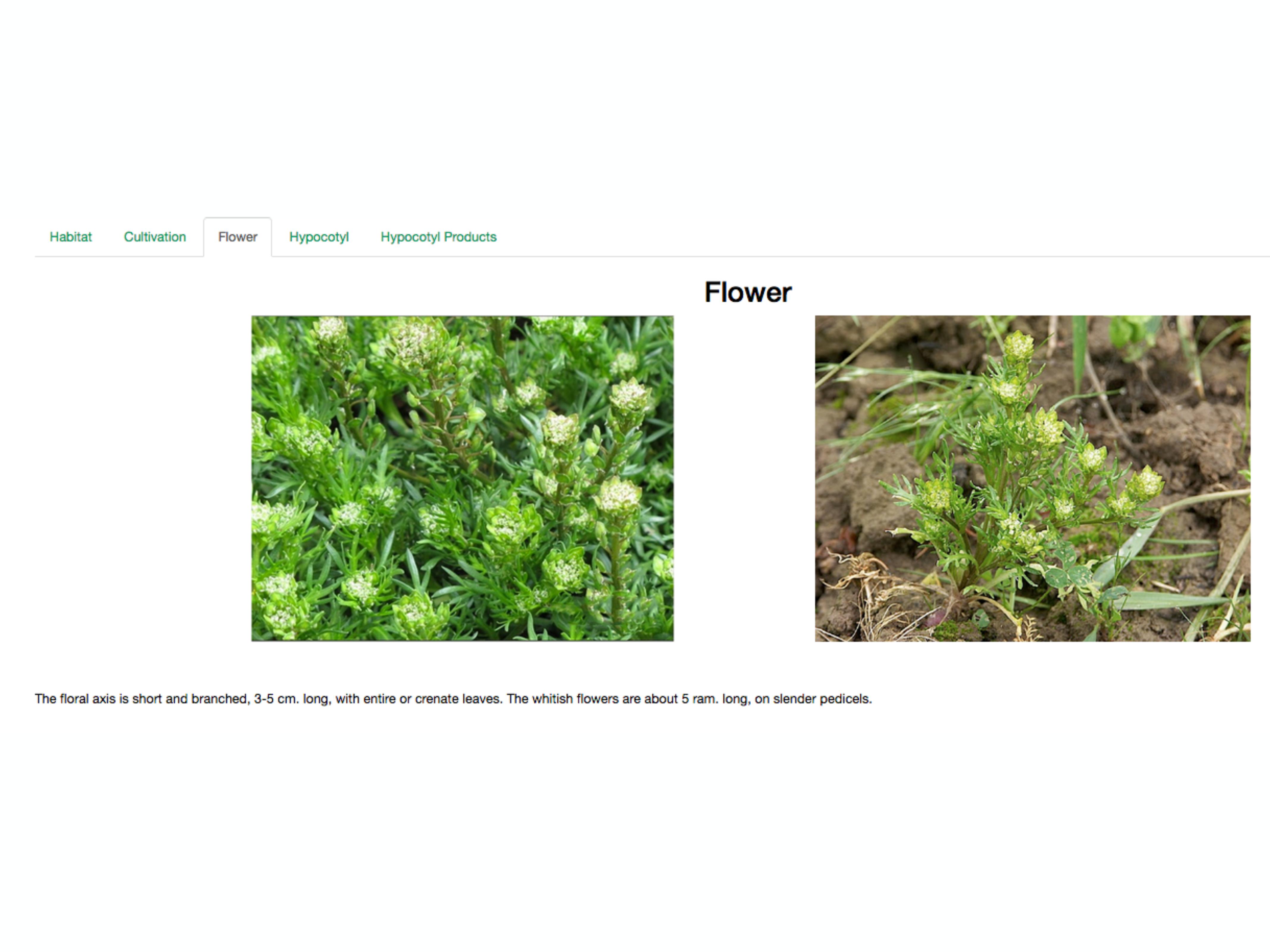

Supplement: Supplementary Data [file bay113_suppl_data.zip › figs9.jpg]
